# Fibril polymorphism affects immobilized non-amyloid flanking domains of huntingtin exon1 rather than its polyglutamine core

**DOI:** 10.1038/ncomms15462

**Published:** 2017-05-24

**Authors:** Hsiang-Kai Lin, Jennifer C. Boatz, Inge E. Krabbendam, Ravindra Kodali, Zhipeng Hou, Ronald Wetzel, Amalia M. Dolga, Michelle A. Poirier, Patrick C. A. van der Wel

**Affiliations:** 1 Department of Structural Biology, University of Pittsburgh School of Medicine, 3501 Fifth Avenue, Pittsburgh, Pennsylvania 15213, USA; 2 Department of Molecular Pharmacology, Groningen Research Institute of Pharmacy, University of Groningen, Antonius Deusinglaan 1, 9713 AV Groningen, The Netherlands; 3 Division of Neurobiology, Department of Psychiatry, Children's Medical Surgical Center, Johns Hopkins University School of Medicine, 600 North Wolfe Street, Baltimore, Maryland 21287, USA

## Abstract

Polyglutamine expansion in the huntingtin protein is the primary genetic cause of Huntington's disease (HD). Fragments coinciding with mutant huntingtin exon1 aggregate *in vivo* and induce HD-like pathology in mouse models. The resulting aggregates can have different structures that affect their biochemical behaviour and cytotoxic activity. Here we report our studies of the structure and functional characteristics of multiple mutant htt exon1 fibrils by complementary techniques, including infrared and solid-state NMR spectroscopies. Magic-angle-spinning NMR reveals that fibrillar exon1 has a partly mobile α-helix in its aggregation-accelerating N terminus, and semi-rigid polyproline II helices in the proline-rich flanking domain (PRD). The polyglutamine-proximal portions of these domains are immobilized and clustered, limiting access to aggregation-modulating antibodies. The polymorphic fibrils differ in their flanking domains rather than the polyglutamine amyloid structure. They are effective at seeding polyglutamine aggregation and exhibit cytotoxic effects when applied to neuronal cells.

## Abstract (web summary)

Huntington's disease is caused by a polyglutamine stretch expansion in the first exon of huntingtin. Here, the authors use infrared spectroscopy and solid-state NMR and show that polymorphic huntingtin exon1 fibres differ in their flanking regions but not their core polyglutamine amyloid structures.

Huntington's Disease (HD) is the most prevalent example of a family of neurodegenerative diseases that have the abnormal expansion of a polyglutamine stretch (polyQ) as their primary genetic cause^1^. HD is a devastating and as-yet incurable disease in which the polyQ expansion occurs within the first exon of the huntingtin protein (htt exon1). As a result of protease activity or missplicing, N-terminal fragments of the mutant protein are generated, including the htt exon1 segment. Misfolding, self-assembly and aggregation of these fragments lead to a gain of toxic function, which ultimately leads to neuronal death. The exact mechanism of toxicity remains uncertain, and different studies report diverging levels of toxicity (or lack thereof) for detectable htt exon1 aggregates, with some reporting an apparent lack of correlation between aggregate burden and toxicity^2^. However, it is increasingly recognized that cells contain different types of aggregates, including also fibrillar aggregates that are not as easily detected as large inclusions^3^,^4^,^5^. Such polymorphism is reminiscent of other amyloids^6^,^7^, and is important, given that the toxicity of htt exon1 aggregates is known to depend on their structure^8^,^9^. Accordingly, toxicity-reducing mechanisms *in vivo* may induce the generation of aggregate species with reduced toxicity^9^,^10^,^11^, in parallel to protein homeostasis and clearance mechanisms that reduce aggregation.

The structural differences that underlie the polymorphism of htt exon1 aggregates remain uncertain. Prior studies have generally attributed them to the expanded polyQ domain, even in cases where low-resolution structural data may not unambiguously distinguish the polyQ and non-polyQ domains^8^,^12^. While the expanded polyQ domain forms the ‘core' of the fibrillar aggregates^13^,^14^,^15^,^16^,^17^, it has become clear that non-polyQ ‘flanking' domains (Fig. 1) have dramatic influences on the misfolding and aggregation pathways of htt exon1 and other polyQ proteins^18^,^19^,^20^,^21^,^22^,^23^. The highly conserved 17-residue N-terminal flanking segment (htt^NT^) is important for the native function of htt, but also initiates and accelerates aggregation of mutant htt exon1 (refs 18, 19, 20, 21). On the other hand, the C-terminal proline-rich domain (PRD) reduces the innate aggregation propensity of the preceding polyQ domain by modulating its conformational ensemble^24^. These flanking domains also are targets for aggregation-modulating post-translational modifications (PTMs), chaperones and antibodies (Fig. 1a)^25^,^26^,^27^,^28^,^29^,^30^,^31^,^32^. However, not all exon1-binding proteins are effective at modulating aggregation. While MW7 and other PRD-binding proteins inhibit aggregate formation and cellular toxicity^28^,^29^,^30^,^33^,^34^, the PRD-binding MW8 antibody does not^30^,^35^.

To understand exon1 aggregate polymorphism, the exon1 aggregation mechanism, and how both can be modulated by htt exon1-binding proteins and PTMs, it is crucial to know the structure of the aggregated species. We have been using magic-angle-spinning (MAS) solid-state NMR (ssNMR) to study mutant htt exon1 and shorter htt-derived peptide fibrils^13^,^15^,^17^,^32^. MAS ssNMR is a powerful tool for elucidating the structure of amyloid fibrils, and is the gold standard for identifying differences among polymorphic amyloid structures^7^,^36^. Mutant htt exon1 fibrils feature a well-defined amyloid core, consisting of polyQ β-hairpins^17^, while the flanking domains lack β-structure^14^,^15^,^16^,^17^. In fibrils formed by synthetic htt N-terminal fragments (HNTFs) that behave similar to full-length exon1 (ref. 37), the htt^NT^ segment features a short amphipathic α-helix^13^,^15^,^32^. Here we refer to these htt^NT^Q_30_P_10_K_2_ peptides (Fig. 1b) as HNTFs. A recent ssNMR study on fibrils prepared using thioredoxin-fused htt exon1 failed to detect the signals for an α-helical htt^NT^, raising the possibility that htt^NT^ has a different structure in fibrillar exon1 (ref. 16). This is an important issue, as the presence of α-helical htt^NT^ provided support for the idea that α-helical htt^NT^ segments play a critical role in exon1 oligomerization and aggregation^13^,^15^,^20^,^21^,^32^. Thus, prior studies of htt exon1 fibrils have been unable to offer a consistent picture of either the detailed structure of the fibrils (and in particular the flanking domains) or the origins of reported fibril polymorphism. Here we report MAS ssNMR studies of different fibrils prepared from htt exon1 featuring a disease-relevant 44-residue polyQ domain. We find that the htt^NT^ also in exon1 fibrils contains a partly immobilized α-helix, and probe in detail the structure and dynamics of the C-terminal flanking domain. The polyQ-proximal region of the PRD is immobilized near the amyloid core surface, reducing access to PRD-binding antibodies. An obvious and reproducible temperature-dependent amyloid-like fibril polymorphism affecting the cytotoxic aggregates is reflected in detectable changes, not in the polyQ as previously suggested, but rather in the non-amyloid flanking domains.

## Results

### Fibril formation by disease-relevant mutant htt exon1

**Figure 1:**
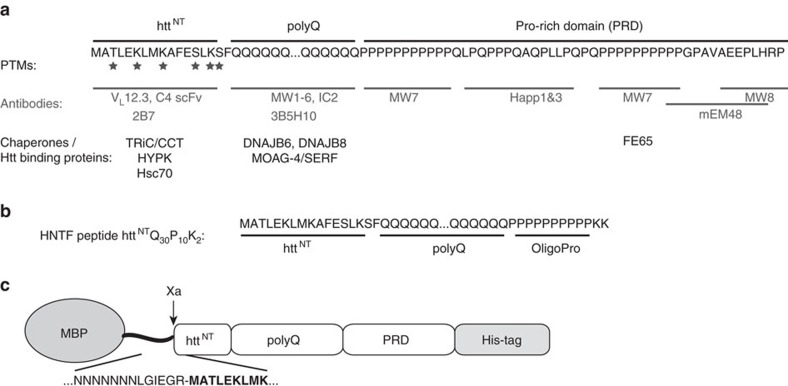
Htt exon1 sequence and domain structure. (**a**) The domain structure and sequence of htt exon1 is shown at the top. The locations of PTMs, as well as the binding sites of various antibodies and other htt-binding proteins are indicated^25^,^26^,^27^,^28^,^29^,^30^,^31^,^32^,^33^,^34^,^58^. (**b**) Design of previously studied^13^ HNTF peptide htt^NT^Q_30_P_10_K_2_. (**c**) Design of the MBP fusion protein, with the sequence of the Factor Xa cleavage site in the linker shown below.

**Figure 2:**
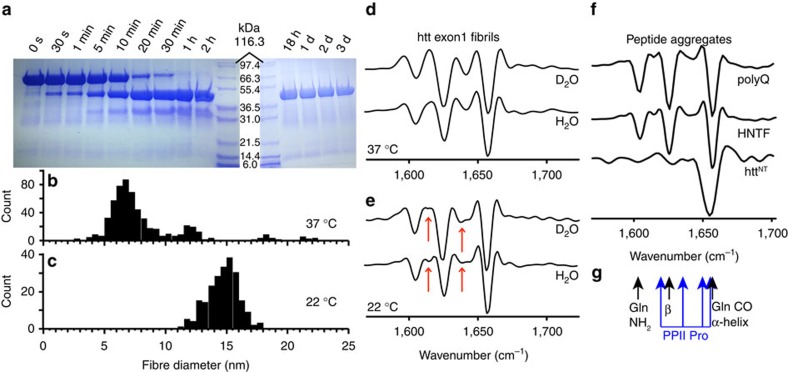
Cleavage and aggregation of mutant htt exon1. (**a**) SDS–PAGE gels showing time-dependent factor Xa cleavage at 22 °C. (**b**,**c**) Fibril width derived from negative-stain TEM on the mature fibrils formed at 37 °C (597 measurements over 99 fibrils) and 22 °C (219 measurements over 73 fibrils). (**d**) Second-derivative FTIR of htt exon1 fibrils formed at 37 °C and (**e**) 22 °C, for fibrils dispersed in either H_2_O or D_2_O. The coloured arrows mark the most notable differences between the fibril types. (**f**) Reference data on fibrillar K_2_Q_31_K_2_, HNTF (htt^NT^Q_30_P_10_K_2_) fibrils, and aggregated α-helical htt^NT^ in PBS buffer, adapted with permission from ref. 13, Copyright 2011 American Chemical Society. (**g**) Resonance frequencies of different secondary structure elements.

For *in vitro* studies, mutant htt exon1 is usually expressed as a fusion protein in which the N terminus of exon1 is fused to a soluble protein tag to inhibit aggregation^38^,^39^,^40^. Cleavage of the linker releases exon1, but commonly leaves behind a non-native N terminus^30^,^38^,^39^,^40^. Such modifications of htt^NT^ can modify the aggregation and toxicity of htt exon1 (refs 8, 18), similar to the effects of htt^NT^ mutations and PTMs^20^,^32^. We therefore generated a fusion construct for htt exon1 with a 44-residue polyQ domain that produces an N terminus just as it is encoded in the genome when the fusion partner is cleaved^15^. A 10-residue linker segment was eliminated from a previously used maltose-binding protein (MBP) fusion protein construct^40^ to yield a new Factor Xa cleavage site (IEGR-MATL) designed to generate the desired 17-residue htt^NT^ (Fig. 1c). To test for correct cleavage, we performed SDS–PAGE and mass spectrometry analyses. Efficient protease cleavage is observed (Fig. 2a), resulting in release of MBP and htt exon1 with the expected molecular masses (Supplementary Table 1 and Supplementary Fig. 1). Thus, even without the extended linker the cleavage site is easily protease-accessible.

Using transmission electron microscopy (TEM) we observed the aggregation of the released htt exon1 (ref. 17). Across a series of independent aggregation trials we observed mature aggregates that appear as elongated unbranched amyloid-like fibrils, but differed specifically in the fibre widths. Consistent with prior work^8^, we find that the temperature at which the aggregation occurs plays a critical role in dictating the fibril morphology. At 37 °C, narrow fibrils are formed (Fig. 2b and Supplementary Fig. 2a,b). Aggregation at 22 °C yields larger fibril widths (Fig. 2c and Supplementary Fig. 2c,d). By Fourier transform infrared spectroscopy (FTIR) both fibril types (Fig. 2d,e) share the same dominant features previously reported for other polyQ, HNTF and htt exon1 fibrils (for example, Fig. 2f)^8^,^13^,^37^,^41^,^42^. The two fibril species display a few differences in their less prominent signals (arrows in Fig. 2e). The partly overlapping resonance frequencies expected for α-helices, β-sheet polyQ amyloid and PPII-helical oligoproline (oligoPro) are indicated in Fig. 2g (refs 13, 37, 41, 42, 43, 44). The differentiating FTIR signals show most similarity to signals expected for the PRD, but an unambiguous assignment is challenging. These polymorphic TEM and FTIR features are reproduced in independently prepared samples (for example, Supplementary Fig. 3).

### SSNMR shows no evidence of polymorphism in the amyloid core

**Figure 3:**
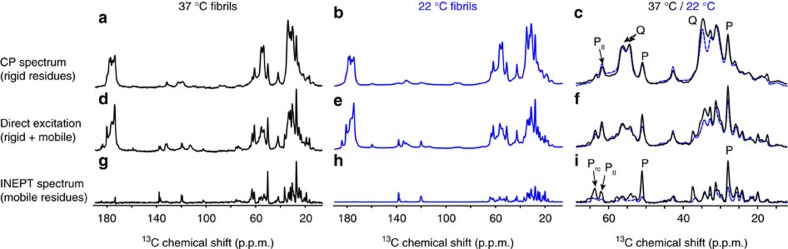
1D ^13^C ssNMR spectra of uniformly ^13^C- and ^15^N-labelled htt exon1 fibrils. (**a**,**d**,**g**) Fibrils were formed at 37 °C, or (**b**,**e**,**h**) 22 °C, and studied using (**a**–**c**) cross-polarization (rigid residues), (**d**–**f**) direct polarization and (**g**–**i**) INEPT-based (mobile residues) MAS ssNMR. (**c**,**f**,**i**) Overlaid aliphatic regions, with assignments indicating the random coil (P_rc_) and PPII-helical Pro (P_II_). The NMR measurements were performed at 275 K on a 600 MHz (^1^H frequency) spectrometer.

**Figure 4:**
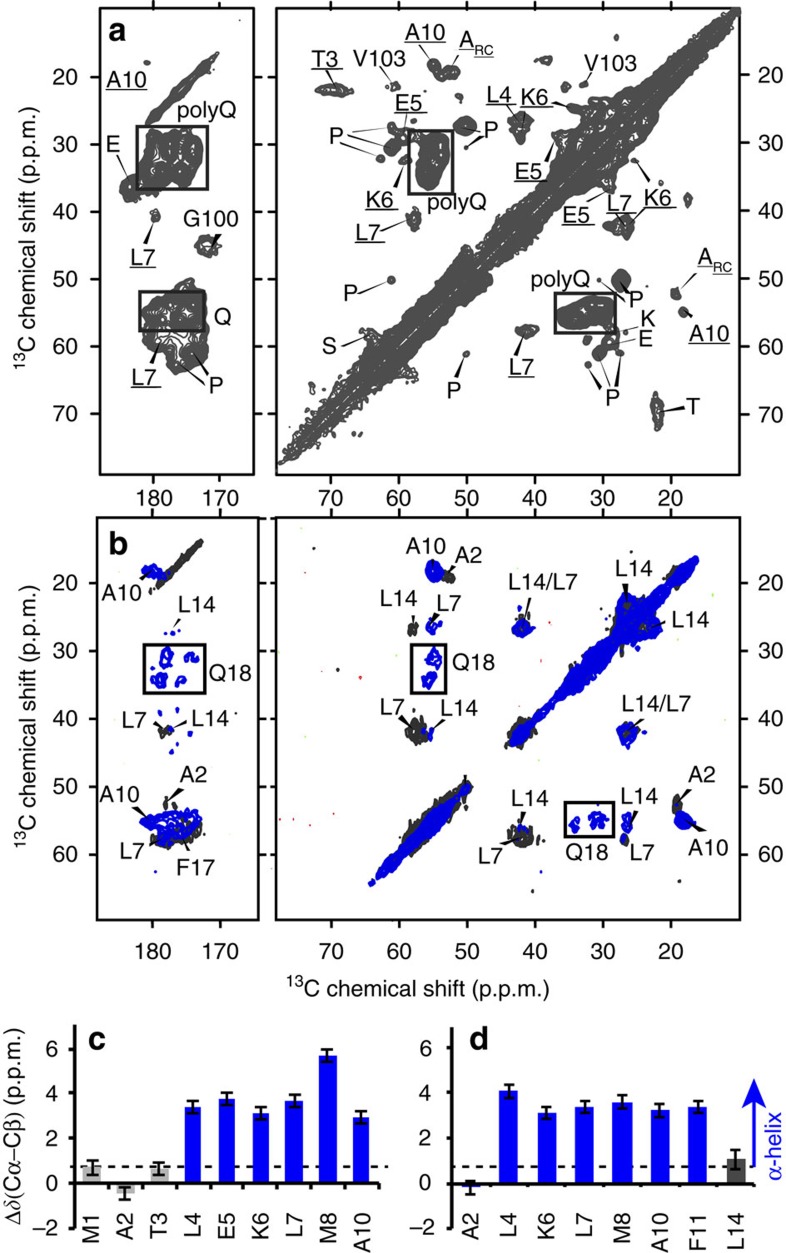
MAS ssNMR identifies the immobilized parts of htt exon1 fibrils. (**a**) 2D ^13^C–^13^C CP/DARR spectrum on U-^13^C,^15^N htt exon1 fibrils prepared at 22 °C. Signals from the polyQ domain are boxed, and underlined peak assignments are from residues in htt^NT^. (**b**) Analogous 2D spectra of HNTF fibrils with site-specific U-^13^C,^15^N-labelling of the indicated htt^NT^ residues and the first Gln of the Q_30_ repeat (Q18)^13^. The blue and black contours are for samples labelled in residues A10/F11/L14/Q18, or A2/L7/F17, respectively. (**c**,**d**) Detection of α-helical secondary structure (blue bars) in htt^NT^ residues of htt exon1 (**c**) and HNTF (**d**) fibrils, based on the secondary chemical shifts Δ*δ*(Cα–Cβ). Error bars reflect the s.d. in the chemical shift (see Supplementary Table 3). The NMR measurements were performed at 267–275 K on a 600 MHz (^1^H) spectrometer. Panel (**b**) was adapted with permission from ref. 13, Copyright 2011 American Chemical Society.

To analyse the fibril structure in more detail, we applied MAS ssNMR to uniformly ^13^C and ^15^N (U-^13^C,^15^N)-labelled htt exon1 fibrils (see Methods and Supplementary Table 2 for experimental details). To identify the rigid domains we use experiments reliant on cross-polarization (CP) and other dipolar-coupling-based transfers, which filter out highly mobile residues. Figure 3a–c compares the 1D ^13^C CP spectra of htt exon1 fibrils prepared at 22 and 37 °C. The spectra are essentially indistinguishable, with no indication of substantial structural differences in the immobile parts of the fibrils. We gain more insights into these rigid domains using two-dimensional (2D) spectra that afford site-specific resolution and thus assignment of residues or residue types (see Supplementary Table 3). Figure 4 shows a 2D ^13^C–^13^C spectrum obtained with CP and dipolar-assisted rotational resonance recoupling (CP/DARR). This spectrum is dominated by signals from the polyQ amyloid core (boxed), with its highly characteristic resonance frequencies^13^,^15^,^16^,^17^,^45^,^46^. A detailed analysis of 2D spectra for the htt exon1 fibrils prepared at 22 and 37 °C (refs 15, 17) reveals no detectable differences in terms of the Gln chemical shifts, relative peak heights, cross-correlation patterns or dynamics (see also below). This stands in contrast to polymorphic Aβ and α-synuclein fibrils that are easily distinguished by their ssNMR spectral differences indicative of distinct amyloid core structures^7^,^36^, as well as prior reports of significant structural changes affecting the polyQ core itself^8^,^12^.

### Flanking domains feature immobilized α- and PPII helices

The 2D CP/DARR spectrum also includes peaks (underlined labels in Fig. 4a) that reproduce the α-helical htt^NT^ signals seen previously in the HNTF fibrils (Fig. 4b)^13^,^15^. The observed resonance frequencies identify a localized α-helix, based on the dependence of Cα and Cβ chemical shift *δ* on the backbone structure. In Fig. 4c,d, this is visualized as positive ‘secondary shifts,' Δ*δ*(Cα-Cβ), for the α-helix spanning residues 4–11 of htt^NT^ (blue bars). The one-dimensional (1D) and 2D CP spectra also feature peaks from the PRD. The dominant PRD signals in Fig. 4a are for Pro residues with chemical shifts characteristic of PPII helices, as previously seen in HNTF and htt exon1 fibrils^15^,^16^. A weaker, but still strong, second set of Pro signals is observed with chemical shifts resembling those of Pro in intrinsically disordered proteins (IDPs), indicating a random-coil-like (Pro_RC_) structure^47^. ^13^C direct-excitation spectra indicate an ∼2:1 ratio of the two populations of Pro residues, independent of the fibril formation temperature (Fig. 3d–f and Supplementary Fig. 4). Other, non-Pro, PRD signals are visible, including peaks for the unique Gly and Val residues G100 and V103. The fact that these htt^NT^ and PRD residues are visible in CP-based spectra implies that both flanking domains are partly immobilized by interactions with the amyloid core or with each other.

### Restricted motion of the polyQ-proximal flanking segments

**Figure 5:**
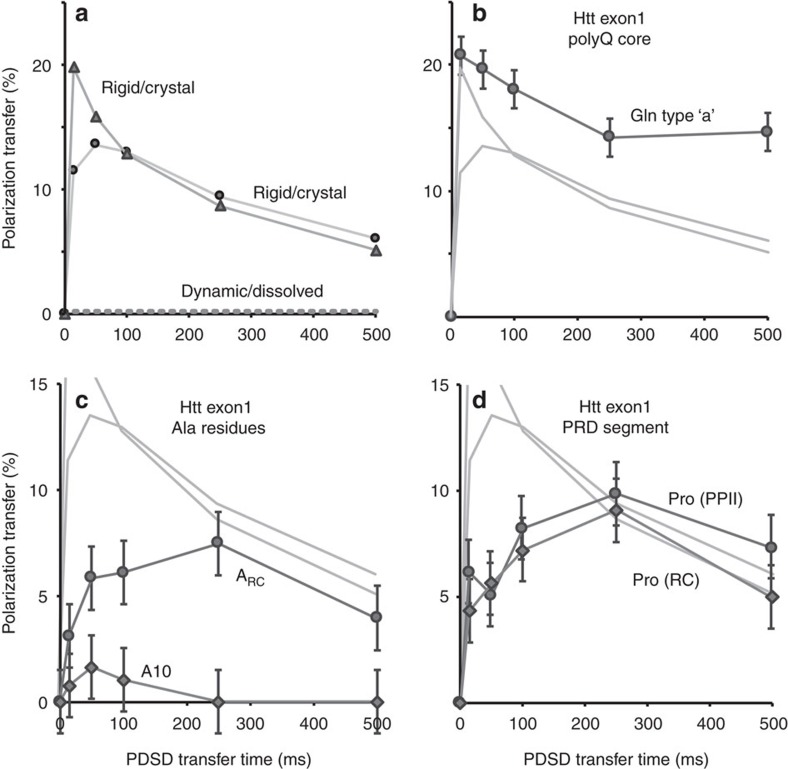
SSNMR dipolar recoupling curves reveal exon1 fibril domain motion. (**a**) Reference PDSD buildup profiles for one-bond Cα–Cβ cross-peaks of the crystalline dipeptide N-acetyl-Val-Leu, reflecting an example of a fully rigid molecule. The dashed line illustrates the lack of buildup for a fully mobile (for example, dissolved) molecule. Intermediate motion is expected to lead to build-up curves in-between these extremes. (**b**) PDSD buildup profiles for Cα–Cβ peaks of type-‘a' Gln in the polyQ core, (**c**) α-helical A10 in htt^NT^, random coil Ala (A_RC_) and (**d**) the random coil (RC) and PPII-structured Pro in the PRD of htt exon1 fibrils formed at 22 °C. Pale grey lines show the reference curves from **a**. Error bars indicate the s.d., as described in the Methods. The NMR measurements were performed at 275 K on a 600 MHz (^1^H) spectrometer.

CP-visible residues may be immobilized, but can nonetheless feature significant and detectable dynamics^15^. Evidence of such dynamics was obtained in a series of ^13^C–^13^C recoupling experiments with proton-driven spin diffusion (PDSD) times of 0–500 ms (Supplementary Fig. 5a–f). The transfer or buildup of polarization (or signal) in such experiments is dependent on dipolar couplings between nearby ^13^C and ^1^H atoms. Dynamics cause an apparent reduction in these couplings, leading to slower and weaker polarization transfer profiles as illustrated in Fig. 5a. In a rigid crystalline peptide, directly bonded Cα–Cβ carbons show a fast polarization buildup that reaches a 10–20% polarization transfer within the first 10–20 ms (solid lines). Some variations in the polarization transfer are typical of the complex mechanism underlying PDSD recoupling^48^. Intermediate timescale molecular motion reduces the effective dipolar couplings and increases relaxation, causing a reduction in both the transfer rate and the transfer maximum^15^. Fully dynamic molecules, such as those in solution, experience complete averaging of the dipolar couplings and, therefore, a lack of ^13^C–^13^C transfer (dashed line). Thus, these PDSD buildup profiles can be used to detect dynamics.

In the exon1 fibrils, we observe a fast buildup and high maximum for Cα–Cβ peaks of the amyloid core Gln (Fig. 5b) that indicates a crystal-like rigid structure. Small variations among the curves are most likely explained by the complex PDSD mechanism rather than changes in dynamics. Much larger changes are seen in Fig. 5c, which shows the buildup curves for the flanking domain Ala signals. Compared to the Gln, the buildup is slower and the attained maximum is much reduced. These dramatic differences in the PDSD buildup curves can only be explained by motion-induced reductions of the dipolar couplings and increases in relaxation. In the PRD, we see that both types of Pro (PPII and random coil) have one-bond signal transfer (Fig. 4d) that is both lower and slower than that of the amyloid core (Fig. 4b). Two-bond Cα–Cγ transfers reveal a difference between IDP-like and PPII Pro (Supplementary Fig. 5g), which indicates that the former undergo increased dynamics. As a secondary probe of these dynamics, we also measured the motional averaging of ^1^H–^13^Cα dipolar couplings for the Pro and Gln residues, using a dipolar-chemical shift (DIPSHIFT) experiment^15^,^49^. The results of these experiments match the results of the PDSD-based measurements. Unlike the dipolar oscillations of the rigid Gln backbones, the Pro residues experience attenuated ^1^H–^13^C dipolar couplings (Supplementary Fig. 6). The PRDs of fibrils formed at 22 °C appear to be slightly more dynamic compared to the 37 °C fibrils. Thus, Pro residues in the flanking domains in htt exon1 fibrils have an intermediate timescale dynamic behaviour similar to those of the htt^NT^ α-helices^15^.

### Variable dynamics of the PRD flanking domains

**Figure 6:**
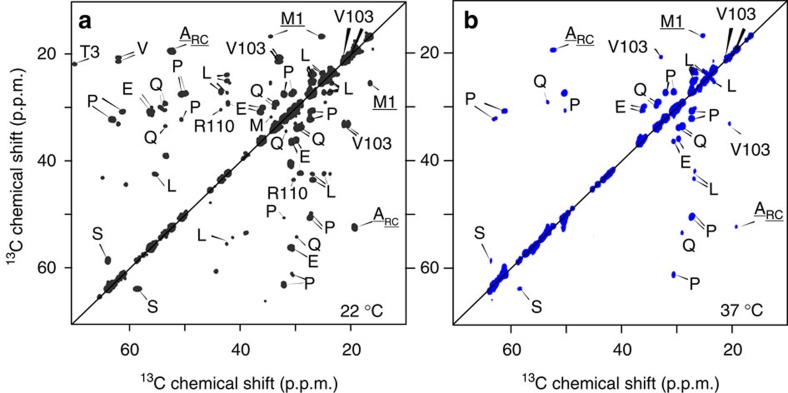
Dynamic residues in the polymorphic htt exon1 fibrils identified via 2D INEPT-based ssNMR. ^13^C–^13^C INEPT/TOBSY spectra for fibrils prepared at 22 °C (**a**), and 37 °C (**b**). Observed residue types are from the very C-terminal tail of the PRD, indicating that this part of the fibrillized protein is highly dynamic. Spectra acquired at 600 MHz (^1^H) and 8.33 kHz MAS, at a temperature of 275 K.

CP-based ssNMR spectra of the polymorphic fibrils showed no chemical shift differences in the polyQ amyloid core or immobilized parts of the flanking domains; however, there is evidence of motional differences in the latter. To test for differences in more dynamic parts of the fibrils, we turn to a different set of motion-sensitive ssNMR experiments. Solution-NMR-like INEPT (Insensitive Nuclei Enhanced by Polarization Transfer) spectra require nuclei to experience slow ^1^H T_2_ relaxation in absence of ^1^H–^1^H decoupling, and thus show only residues with very high mobility. Both the 22 and 37 °C fibrils yield peaks in INEPT spectra (Fig. 3g–i). Strikingly, we observe a lot more signal for fibrils formed at 37 °C compared to those obtained at 22 °C. Both exon1 samples differ from htt^NT^Q_30_P_10_K_2_ fibrils, which show no INEPT signals, suggesting that the dynamic residues are in the latter part of the PRD that is missing from such peptides^13^,^15^,^16^. In 2D ^13^C–^13^C INEPT/TOtal through Bond correlation Spectroscopy (TOBSY) spectra for both fibril types (Fig. 6) peaks are observed that differ from those in the 2D CP-based spectra (see overlay in Supplementary Fig. 7), with a few exceptions discussed below. On the basis of the 2D data, we can assign the mobile residues or residue types, as marked in Figs 3 and 6. The strongest peaks are from Pro residues (for example, Fig. 3i), with other peaks reflecting residue types that are only present in the tail end of the PRD: Val103, E105, E106 and Arg110. The chemical shifts indicate that this highly mobile C-terminal tail is unstructured.

As noted, the efficiencies of CP and INEPT-based spectra have opposite dependencies on molecular motion. Mobile residues visible as strong peaks in INEPT spectra are expected to be missing from CP-based spectra. A residue with a prominent CP signal, in contrast, must be in some way immobilized, have fast T_2_ relaxation and thus be invisible in INEPT spectra. Seeing substantial peaks from the same residue in both spectra (Supplementary Fig. 7) implies that such a residue is present in different subpopulations that have markedly different mobilities. Intriguingly, this applies to many of the PRD signals: the PPII Pro, the IDP-like Pro, as well as V103. Overall, ∼2/3 of the Pro are in PPII structure (Supplementary Fig. 4a,b). However, among the INEPT-visible mobile prolines it is the IDP-like peaks that are dominant (Supplementary Fig. 4c). We conclude that different molecules throughout the sample have PRDs with different degrees of flexibility. When comparing the fibril polymorphs, in the 37 °C fibrils the population of proteins with dynamic PRDs is notably larger, even though the overall ratio of PPII to unstructured Pro remains the same (Supplementary Fig. 4a,b).

### Polymorphic differences in PRD motion and accessibility

**Figure 7:**
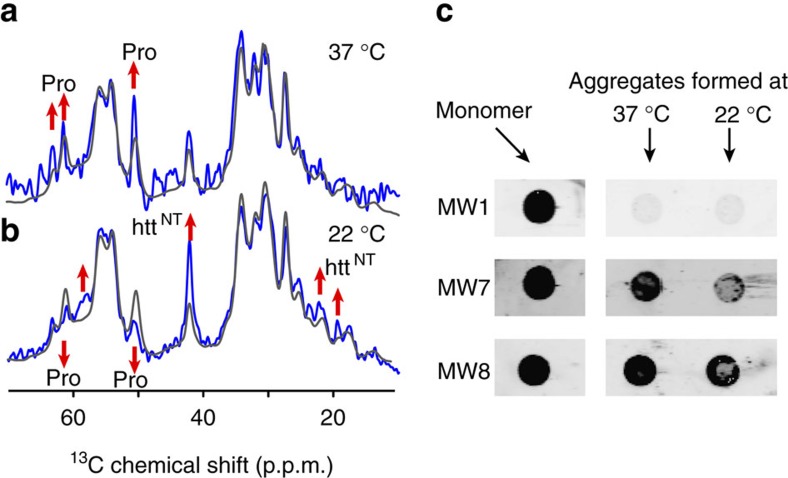
Accessibility of the htt exon1 fibril PRDs probed by solvent-filtered ssNMR and antibody binding. (**a**,**b**) Solvent accessibility of htt exon1 fibrils prepared at (**a**) 37 °C and (**b**) 22 °C probed by ssNMR. Peak intensities after 7 ms ^1^H–^1^H diffusion from the solvent into the fibrils (blue) are compared to the ^13^C CP spectrum in absence of T_2_-based solvent filtering (grey). Each spectrum was normalized to the highest peaks to highlight the relative solvent exposures. Up/down arrows indicate sites with high/low solvent accessibility. The NMR measurements were performed at 275 K on a 600 MHz (^1^H) spectrometer. (**c**) Dot blot analysis shows that in the monomeric protein the polyQ domain, oligoPro segments and PRD tail are all accessible for binding by MW1, MW7 and MW8, respectively (Fig. 1). Upon aggregation at 22 or 37 °C, MW1 binding to the polyQ is largely abolished, while the PRD tail is still strongly recognized by MW8. OligoPro binding by MW7 is weaker in the 22 °C fibrils compared to the 37 °C polymorph.

As protein dynamics often correlate to solvent interactions, we submitted both fibril forms to ssNMR measurements that can evaluate solvent exposure in a residue-specific manner. The employed experiments first eliminate the ^1^H–^13^C CP signals generated from proteinaceous hydrogens by filtering out the latter based on their faster T_2_ relaxation. Then, we detect the signal recovery as a function of time-dependent transfer of solvent ^1^H polarization into the fibril. Residues that are most solvent-exposed recover the fastest, while those that are buried take longer to re-appear. Figure 7a,b compares the overall, unfiltered CP signal (grey line) to the partly repolarized fibril signal after 7 ms of ^1^H–^1^H diffusion (blue), with both spectra normalized to their maximum peaks. In the 37 °C fibrils the highest signal recovery is seen for Pro residues, indicating a high degree of solvent accessibility for the PRDs. The repolarization is fastest for the IDP-like Pro, consistent with their higher mobility. The data for the 22 °C fibrils are different, in that the polarization transfer to the PPII Pro trails that of other parts of the fibrils. Thus, in these samples the PPII helices have a surprisingly reduced solvent accessibility, consistent with their more restricted motion. Conversely, the signals from htt^NT^ are notably enhanced (upon 7 ms ^1^H–^1^H transfer) compared to the 37 °C fibrils, reminiscent of the htt^NT^ α-helix in HNTF fibrils^13^,^15^. Thus, the flanking domains in these polymorphic fibrils feature correlated differences in their dynamics and solvent exposure.

### Occlusion of the PRD domains limits antibody access

Next, we examined a number of biochemical or functional characteristics of the htt exon1 fibril polymorphs. First, dot blot experiments were used to probe the domain-specific accessibility of the fibrils to htt-exon1-specific antibodies^35^ (Fig. 7c). In line with prior reports^30^,^35^, exon1 fibril formation causes region-specific reductions in the binding of antibodies to their epitopes. PolyQ-binding MW1 antibodies bind monomers, but have very low affinity for fibrils (Fig. 7c), because of complete sequestration of their epitopes^30^. The epitopes of the MW7 and MW8 antibodies are in the oligoPro segments and C-terminal PRD tail, respectively (Fig. 1a)^35^. MW8 binds to the PRDs of our mature exon1 fibrils, with an efficiency that is similar to the unaggregated protein (Fig. 7c). MW7 shows a reduced affinity to the aggregates, with the largest reduction in binding seen for the wider 22 °C fibrils, which suggests an increased sequestration of its epitopes in those aggregates. These findings are consistent with prior studies that probed aggregated mutant htt exon1 with these antibodies, both *in vitro* and as cellular inclusions^30^,^35^.

### Seeding activity and cytotoxic effects of the polymorphs

**Figure 8:**
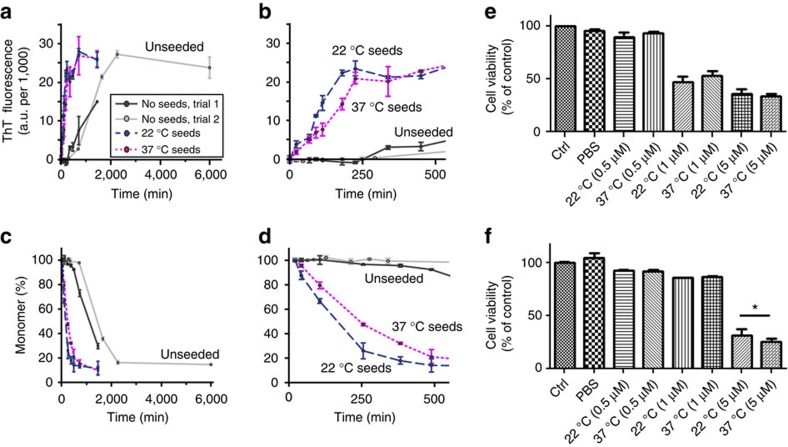
PolyQ protein recruitment and neuronal toxicity assay results. (**a**) Aggregation kinetics at 22 °C in the absence (solid black and grey lines) and presence (dashed lines) of pre-made seed aggregates, detected as ThT fluorescence at indicated time points after complete trypsin cleavage of the htt exon1 fusion protein. Dark blue and magenta dashed lines reflect the aggregation in presence of 20 mol-% htt exon1 aggregates formed at 22 and 37 °C, respectively. The unseeded reactions have lag phases exceeding 4 h, which are eliminated by the seeds. Error bars indicate s.d., with *n*=2–3, as described in the Methods section. (**b**) Enlargement of the first 500 min. (**c**,**d**) Results of a single (*n*=1) HPLC measurement of the residual monomer concentration after aggregate sedimentation, applied to the same samples, as a complementary measure of aggregation. Error bars reflect the estimated peak integration error as described in the Methods. (**e**) Cellular viability of human dopaminergic neuronal cells upon exposure to varying concentrations of pre-formed fibrils prepared at 22 and 37 °C. The data reflect MTT reduction assays performed after 24 h (*n*=2; two biological replicates with three technical replicates each—shown is the mean with s.d. compared to non-treated controls set at 100%). (**f**) Cell viability assay data for a 24 h exposure of immortalized HT-22 neurons (*n*=2; two biological replicates with 6 technical replicates each–shown is the mean with s.d. compared to non-treated controls set at 100%; *P<0.05, Mann–Whitney non-parametric test).

We also compared the seeding activity of two polymorphs using a previously reported seeding assay^50^. Figure 8 shows the results of this assay in which aggregation at 22 °C in presence and absence of 20 mol-% of pre-aggregated seeds was monitored by thioflavin T (ThT) fluorescence and HPLC-based monomer concentration measurements. In absence of seeds, aggregation (after trypsin cleavage) initiates with a lag phase that exceeds 4 h (Fig. 8b,d). The presence of seeds abolishes the lag, leading to a significant decrease in the half time of aggregation. This seeding ability affects expanded polyQ proteins (Fig. 8) and shorter peptides with a 23-residue polyQ domain (Supplementary Fig. 8).

To probe for potential cytotoxic effects, we exposed two neuronal cell types to micromolar concentrations of each polymorph. Two different neuronal cell types were tested: an immortalized murine hippocampal neuronal cell line and human differentiated dopaminergic neurons^51^,^52^,^53^. Fibrils were applied at different concentrations (0.5, 1 and 5 μM). The low concentration of 0.5 μM did not induce any cell death in either of the neuronal cell types, while we detected differential neuronal toxicity with 1 μM that induced dopaminergic cell death but not in immortalized neuronal cells. At higher concentrations, fibrils promoted neuronal cell death in both cell types. Our results suggest that human dopaminergic neurons are more susceptible to this external stimulus since neuronal death occurred at lower fibril concentrations (Fig. 8e,f). Across independently performed assays, some variability was observed, but most of the data indicate a slightly larger impact from the thinner fibrils formed at 37 °C. One indicator of this difference is also seen in the treated neurons' morphology, which is noticeably changed by the fibrils formed at 37 °C, but not by those prepared at 22 °C (Supplementary Fig. 9). Thus, both fibril polymorphs are biologically active, with the *in vitro* seeding assays and cytotoxicity measurements indicating a modest, but nonetheless detectable, difference between the two polymorphs.

## Discussion

We prepared amyloid-like fibrils from mutant htt exon1 that lacked undesirable modifications of its crucial htt^NT^ segment, and studied the fibril structure and how it depends on the fibrillation temperature. At 22 and 37 °C we obtained amyloid-like fibrils with different widths (Fig. 2), of 15 and ∼6 nm, respectively. When examined by FTIR, specific differences were apparent in smaller signals present alongside the invariant dominant polyQ core signals (Fig. 2d,e). The fibrils' highly rigid polyQ domains also featured the same characteristic ssNMR signature^46^. In prior work on these same htt exon1 fibrils^15^,^17^, we used ssNMR to reveal ∼20-residue-long β-strands forming a β-hairpin structure within the aggregated polyQ domain and used *in silico* analysis to show that alternative polyQ models have distinct ssNMR spectral signatures.

By CP-based ssNMR we observed signals from the htt^NT^ α-helix previously observed in HNTF peptide fibrils^13^,^15^,^32^, showing its presence in full-length mutant htt exon1 fibrils. The exon1 htt^NT^ helix experiences significant dynamics that reflect molten-globule-like dynamics also seen for α-helical htt^NT^ in HNTF fibrils^15^. These dynamics reduce ssNMR peak intensities and may in part explain why previously published ssNMR spectra of mutant htt exon1 fibrils failed to show signal from the htt^NT^ (refs 15, 16). It is also possible that the exon1 aggregation process was modulated by residual htt^NT^-attached linker residues^16^, which are avoided in our exon1 and HNTF constructs with unmodified 17-residue htt^NT^ segments. Our observation of α-helical structure in the htt^NT^ of fibrillar exon1 lends further support to the idea that α-helical htt^NT^ interactions play key roles in the htt exon1 aggregation mechanism. Htt^NT^ is thought to initiate and accelerate aggregation via the formation of htt^NT^-htt^NT^ α-helical bundles^13^,^25^,^41^. Flanking domain interactions play similarly important roles in the aggregation pathways of other polyQ disease proteins^22^,^23^.

**Figure 9:**
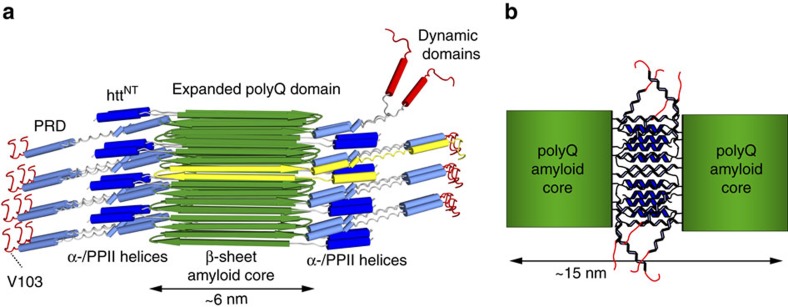
Schematic proposed model of htt exon1 fibrils. (**a**) The htt^NT^ α-helices (dark blue) and PRD PPII helices (light blue) are immobilized and tightly clustered on the perimeter of the rigid amyloid core (green β-strands). C-terminal domains show increased dynamics, either in the form of the unstructured C-terminal tail or a subpopulation of more exposed PRDs (top right; red). An individual protein monomer with its β-hairpin-based polyQ core is shown with lighter (yellow) β-strands. (**b**) Schematic illustration of interfilament flanking domain interactions that we propose to explain the larger TEM-based widths of the fibrils formed at 22 °C, as well as the observed differences in accessibility and immobilization of the PRD.

The most notable ssNMR signals for the PRD are from prolines, present in both PPII and IDP-like random-coil structures. Their relative populations, estimated from direct-excitation ^13^C spectra, appear to be 2:1 independent of the fibril formation temperature (Supplementary Fig. 4a,b). The PPII structure is likely due to the two oligoPro segments of the PRD (Fig. 1a), of which ssNMR previously showed the first to adopt a PPII structure in HNTF fibrils^13^,^15^. The 2:1 intensity ratio shows that the remaining 10 Pro of the PRD do not form stable PPII helices. Perhaps surprisingly, this is not accompanied with IDP-like dynamics, given that the IDP-like Pro are visible in CP spectra. The PDSD and DIPSHIFT experiments indicate similar dynamics for both types of CP-detected Pro residues (Fig. 4). Interestingly, significant CP-based signals are seen for residues up to V103. Thus, these parts of the PRD that do not occupy regular secondary structure are, nonetheless, not free to move around. We attribute this lack of motion to intermolecular interactions due to clustering of PPII- and α-helical flanking domains^23^,^54^. Such interactions also explain the reduced binding by the MW7 antibody (Fig. 7c), while the C-terminal tail is flexible and accessible for strong MW8 binding (Fig. 7c). Thus, are data reveal a transition from a polyQ-proximal semi-rigid PRD segment to a highly dynamic flexible tail, with an apparent transition point at or near residue V103. This is sketched schematically in Fig. 9a, with the mobile C-terminal tail segments shown top right in red. The immobilized PRD segments are not as rigid as the β-sheet polyQ amyloid core, as they experience dynamics similar to those of the htt^NT^ α-helices^15^.

The INEPT-based ssNMR spectra also contain signals from both IDP-like and PPII-helical Pro, indicating the presence of exposed and highly dynamic PPII helices in a subset of the protein molecules in the sample. The HNTF fibrils without C-terminal PRD segments lack such INEPT signals. We speculate that the first oligoPro segment is typically immobilized in its location directly attached to the immobile polyQ core (see ref. 15), while the mobile segments are in the latter part of the PRD (top right of Fig. 9a). Those exon1 monomers with the more mobile PRDs should be more accessible to interacting proteins, including the MW7 antibody (Fig. 7c).

Prior studies have indicated polymorphism in aggregates formed by htt exon1 and other N-terminal fragments, with corresponding effects on the aggregates' cellular toxicity^4^,^8^,^9^,^12^. Both in our hands (Fig. 2) and in earlier work^8^, FTIR indicated structural differences between exon1 fibrils made at different temperatures. By ssNMR we observe differences not in the polyQ domain, but rather in the other exon1 domains. For instance, the PRD domains of 37 °C fibrils have a larger proportion of highly flexible (Fig. 3i) and solvent-exposed (Fig. 7) residues. Conversely, Pro residues in 22 °C fibrils are more restricted in their motion (Fig. 3) and less solvent-accessible (Fig. 7). Thus, unlike prior studies^8^,^12^, we find the polymorphism to be predominantly reflected in the dynamics and accessibility of the non-amyloid flanking domains.

As discussed previously^15^, fibrillar htt^NT^ α-helices are stabilized by intermolecular interactions that immobilize them enough to render them visible by CP ssNMR. These htt^NT^ interactions are important for the oligomerization of htt exon1 and contribute to the stability of the fibrils^32^. We propose that in the mature fibrils flanking domain interactions similarly sequester and immobilize the PRD domains, and thus limit accessibility and binding by proteins for the most Pro-rich parts of the PRD—for example, the MW7 antibodies (Fig. 7c). The origins of this are found in the polyQ amyloid core. SSNMR studies of aggregates formed by polyQ-expanded exon1 and polyQ peptides note that polyQ amyloid contains long antiparallel β-strands with few turn regions^17^,^45^. In our mutant htt exon1 fibrils with 44-residue polyQ domains, we observe 90% of the residues in the β-sheet parts of β-hairpins, separate from the ∼10% of polyQ residues that form the single intervening β-turn^17^. The intramolecular β-hairpin places flanking domains in close proximity to each other in the assembled fibril, limiting their freedom of motion and accessibility (Fig. 9). The polyQ-proximal secondary structure elements are further constrained by the short linkers that connect them to the rigid amyloid core^13^,^15^. Thus, the β-sheet fibre core is surrounded by sterically constrained and densely packed α- and PPII-helical flanking domains, in contrast to reports that the PRD is primarily dynamic^14^,^16^. Figure 9a shows a schematic structural model designed to illustrate the relative dimensions of the flanking domains and an amyloid core featuring 20-residue β-strands. The latter part of the PRD is likely only weakly immobilized in a single, ∼6 nm-wide, filament. We hypothesize that the structural and motional features of the ∼15 nm-wide 22 °C fibrils are most easily explained by flanking domain interactions tying together two filaments, as illustrated in Fig. 9b. This would intertwine the polyQ-proximal flanking segments of the filaments through additional interactions among the α- and PPII helices^54^. Nonetheless, as detected in PDSD and DIPSHIFT experiments, these flanking domains retain molten-globule-like dynamics that greatly reduce the dipolar coupling constants and thus limit ssNMR sensitivity and complicate long-range distance measurements.

Both fibril polymorphs have a strong seeding ability that affects both expanded and non-expanded polyQ aggregation (Fig. 8 and Supplementary Fig. 8). Intriguingly, a small, but seemingly significant, difference is observed between the two polymorphs. This difference in seeding activity shows opposite trends for the two different target peptides, which precludes a straightforward explanation in absence of further studies. Both fibril polymorphs were also shown to have cytotoxic and morphological effects when provided extracellularly to neuronal cells, with a more visible impact by fibrils formed at 37 °C. On the basis of the available data, it remains unclear what dictates the differences in seeding ability and toxicity, and whether or how these two activities may be related. In terms of the cellular impacts, it is likely that a key determinant relates to the ability of fibrils to be taken up, which may depend on the fibril stability and width^8^,^55^,^56^. The exposure of htt^NT^ and PRD domains may also be significant as they modulate interactions with cellular membranes, which may also affect cellular uptake and cytotoxic membrane disruption^57^.

Thus, our results point to differences in the flanking domains' exposure and interactions as being important in htt exon1 aggregates' structure and function. Factors that modulate flanking domain interactions are known to affect cellular toxicity. In htt^NT^, these factors include covalent PTMs and non-covalent binding by chaperones and antibodies^25^,^32^,^58^. The intimate interactions of the polyQ-proximal PRD segments in the fibrils rationalize the finding that also PRD-binding proteins interfere with exon1 aggregation, unless they bind the disordered tail^28^,^29^,^30^,^33^,^34^. Such PRD-based aggregation-inhibiting effects are harder to reconcile with a fibril model in which the entire PRD is flexible and exposed^14^,^16^. An intriguing question is how flanking domain arrangements may affect cellular toxicity, resistance to clearance mechanisms, membrane interactions or fibrils' ability to sequester other proteins^59^,^60^. Further structural studies will be critical to gain a complete understanding of exactly how htt exon1 aggregate structure, stability and toxicity are correlated.

## Methods

### Protein expression and purification

The plasmid encoding mutant htt exon1 with 44 consecutive glutamine residues was modified from a MBP-fusion construct described previously^40^. A single-step deletion mutagenesis reaction using the QuikChange II XL site-directed mutagenesis kit (Agilent Technologies, Santa Clara, CA, USA) was used to remove from the MBP–exon1 linker region 10 amino acids (ISEFGSMSTGGG), which would otherwise remain attached to the exon1 N terminus following Factor Xa cleavage. The employed primer sequences were 5′-CAACCTCGGGATCGAGGGAAGGATGGCGACCCTGGAAAAGCTTATG-3′ and 5′-CATAAGCTTTTCCAGGGTCGCCATCCTTCCCTCGATCCCGAGGTTG-3′. The htt exon1 construct was codon-optimized by GenScript (Piscataway, NJ), using the OptimumGene algorithm for expression in *Escherichia coli* (yielding 5′-AGTAGCAACAATAATAATAATAACAACAACAACAACCTGGGTATCGAAGGCCGTATGGCAACGCTGGAAAAACTGATGAAAGCATTTGAATCCCTGAAAAGTTTCCAGCAGCAACAACAACAGCAACAGCAGCAGCAGCAGCAACAGCAGCAGCAACAACAGCAGCAACAACAGCAACAGCAACAACAACAACAGCAGCAACAGCAACAACAACAGCAGCAGCAGCAACAGCAACAGCCGCCGCCGCCGCCGCCGCCGCCGCCGCCGCCGCAACTGCCGCAACCGCCGCCGCAGGCGCAACCGCTGCTGCCGCAGCCGCAGCCGCCGCCGCCGCCGCCGCCGCCGCCGCCGGGCCCGGCTGTTGCTGAAGAACCGCTGCATCGCCCGAGTGGCTCCCATCACCATCACCATCAT-3′). This construct was subcloned into the pMALc2x plasmid through the restriction digest sites EcoRI and HindIII.

The fusion protein was overexpressed in *E. coli* BL21(DE3)pLysS cells (Invitrogen, Grand Island, NY). Isotopic labelling followed an optimized isotopic labelling protocol^61^, starting by growing the cells in 1 l LB medium with ampicillin and chloramphenicol at 37 °C and 250 r.p.m. until an optical density (OD_600_) of ∼0.65. The cells were pelleted at 1,677*g* for 15 min, resuspended in 50 ml M9 salt solution lacking nitrogen and carbon sources and then pelleted again at 1,677*g* for 15 min, prior to resuspension in 250 ml M9 media containing U-^13^C-D-glucose and ^15^N-ammonium chloride (Cambridge Isotope Laboratories, Tewksbury, MA). The cells were brought to their fast growth phase by cultivating at 37 °C for 30 min and 250 r.p.m. A temperature ramp from 37 to 18 °C over 30 min at 250 r.p.m. was applied, followed by 30 min at 18 °C, to prepare for induction. Protein expression was induced by adding 0.8 mM isopropyl β-D-thiogalactopyranoside (RPI Corp., Mt Prospect, IL), along with 0.02% (w/v) [^13^C]-D-glucose, 0.01% (w/v) [^15^N] ammonium chloride and 100 μM ZnSO_4_. The fusion protein was overexpressed at 18 °C for 16 h, after which the cells were pelleted for 20 min at 7,000*g*. Cell pellets were stored at −20 °C at least 12 h before lysis. To lyse the cell, cell pellets were thawed on ice for 30 min. Then, the pellets were resuspended in buffer A, which is PBS (pH 7.4) containing 0.02% (w/w) sodium azide that had been sterilized by filtration through a Nalgene Rapid-Flow 500 ml bottle top 0.2 μm filter (Thermo Scientific, Waltham, MA). The resuspended cells were kept on ice, following addition of 1 mM phenylmethanesulfonyl fluoride (ACROS, Fair Lawn, NJ), and 2 mg ml^−1^ lysozyme (Hampton Research, Aliso Viejo, CA). After 40 min., cells were broken by sonication (Misonix Inc., Farmingdale, NY) on ice, applying ∼40 W of sonication for a total of 20 min, alternating 10 s pulses and breaks of 10 s. Cell debris was removed by centrifugation (38,720*g* for 1 h). The soluble fusion protein was purified into PBS buffer over a 50–200 mM imidazole gradient using a nickel column, and then buffer-exchanged into PBS buffer to remove the residual imidazole^15^. Purity and identity were verified by SDS–PAGE (12%) and electrospray ionization time-of-flight mass spectrometry (Genomics and Proteomics Core Laboratories, University of Pittsburgh), which was also used to verify the molecular mass and isotopic labelling (where applicable) of the fusion protein, the cleaved MBP solubility tag and the htt exon1 monomer.

### Fibril formation

The purified fusion protein was buffer-exchanged to buffer A (defined above) using centrifugal filter units (Millipore, Billerica, MA). To release htt exon1, the fusion protein was cleaved by treating with Factor Xa (Promega, Madison, WI) at 22 or 37 °C, as indicated. After addition of 0.55 μg of Factor Xa to a 10 μl 44.7 μM solution of the fusion protein (28.7 μg), the progression of cleavage and aggregation was monitored by SDS–PAGE (Bio-Rad Mini-Protein Precast TGX Gels 12%) and TEM (see below). The full SDS–PAGE gels are also shown in Supplementary Fig. 1. Samples (10.5 μl) were mixed with an equal volume of SDS–PAGE loading dye to terminate the reaction, and then analysed by SDS–PAGE (Bio-Rad Mini-Protein Precast TGX Gels 12%). For MAS ssNMR studies, uniformly ^13^C- and ^15^N-labelled (U-^13^C,^15^N) fusion protein was cleaved and allowed to aggregate over 3 days, after which the labelled htt exon1 fibrils were pelleted down at 3,000*g* for 20 min and resuspended in 1 ml buffer A. MAS ssNMR samples were packed in 3.2 mm MAS ssNMR sample holders (Bruker Biospin, Billerica, MA, and CortecNet, Brooklyn, NY) using a home-built ultracentrifugal sample-packing tool^62^ operated for 1 h at 150,000*g*. The supernatant was discarded, and pelleted fibrils were washed at least three times with buffer A prior to sealing of the MAS rotor.

### Transmission electron microscopy

The fibril morphology and progression of fibril formation by htt exon1 were monitored using negative-stain TEM. Aliquots of sample were diluted with PBS buffer, and then deposited onto freshly glow-discharged carbon-coated copper grids. After removal of excess buffer, grids were treated with negative stain that was adsorbed for 30 s prior to blotting. The 22 and 37 °C exon1 fibrils were stained with 1% (w/v) uranyl acetate and 1% phosphotungstic acid, respectively. The 22 and 37 °C exon1 fibrils in Supplementary Fig. 3 were stained with 1% (w/v) uranyl acetate. Images were obtained at 6,500–30,000-fold magnification using a Tecnai T12 TEM (FEI, Hillsboro, OR) operating at 120 kV and equipped with an UltraScan 1000XP CCD camera (Gatan, Pleasanton, CA). Fibril widths were measured using ImageJ's straight line freehand tool (NIH, Bethesda, MD). Each measurement spanned the length of the negative-stained area of the fibre with similar contrast. Pooled positive stain on the edges of the fibres was not included in the measurements. In images with low resolution, the fibre diameter was determined in regions with the clearest defined boundaries. At least three measurements were obtained per fibre.

### FTIR spectroscopy

FTIR spectroscopy was performed using an MB series spectrophotometer with the PROTA software (ABB Bomem, Quebec City, QC, Canada). Aggregates were harvested by centrifugation for 30 min at 20,817*g* in a tabletop Eppendorf 5415C centrifuge, and pellets washed three times with PBS buffer. Pellets containing aggregates were resuspended in either PBS buffer or deuterated PBS buffer at around 10 mg ml^−1^ concentration and incubated for 24 h. Spectra of the resuspended aggregates were acquired at room temperature by placing the aggregate suspension between two polished CaF_2_ windows using a BioCell module (BioTools Inc.). Data from a total of 400 scans were collected with 4 cm^−1^ resolution at room temperature. Spectra were corrected for residual buffer absorption by subtracting the appropriate buffer-alone spectrum interactively until a flat baseline was obtained between 1,700 and 1,800 cm^−1^. Second-derivative spectra for the amide I region were calculated from the primary spectrum by using the PROTA software.

### Dot blot antibody-binding assays

Identically sized aliquots of samples containing 1 μg of unaggregated MBP-fusion protein or aggregated protein in buffer A were transferred to a nitrocellulose membrane using a Bio-Dot apparatus (Bio-Rad, #170–6,545). Blots were incubated overnight with Odyssey Blocking Buffer (PBS) from LI-COR Biosciences (Lincoln, NE, USA), washed three times with TBST (10 mM Tris-HCl, pH 7.5, 150 mM NaCl, 0.1% (v/v) Tween-20, 0.05% (w/v) sodium azide) and incubated with a 1:5,000 dilution of the appropriate antibodies for 3 h. Two independent dot blot assays were performed. The MW1, MW7 and MW8 antibodies developed in the Patterson lab^35^ were obtained from the Developmental Studies Hybridoma Bank (DSHB), created by the NICHD of the NIH and maintained at The University of Iowa, Department of Biology, Iowa City, IA. As controls, we obtained from the DSHB, 2A12 anti-GASP (deposited by Krasnow, M.A.; DSHB Hybridoma Product 2A12) and anti-glass-bottom boat (GBB 3D5-24; Guillermo Marquéz; University of Minnesota) antibodies against drosophila proteins. After washing with TBST to remove unbound material, blots were incubated for 2 h with a 1:10,000 dilution of Alexa Flour 680 conjugate of anti-mouse IgG (Invitrogen, A21057) and then washed four times with TBST. Blots were visualized using a LI-COR Odyssey Infrared Imaging System (LI-COR Biotechnology, Lincoln).

### Seeding assays

The aggregates' seeding ability was measured using seeding assays^21^,^37^,^50^. As seeding material, mutant htt exon1 fibrils were obtained after 5 days of aggregation at 22 and 37 °C, using starting monomer concentrations of 0.2 mg ml^−1^ (15.5 μM) and 0.14 mg ml^−1^ (10.1 μM), respectively. The aggregates were pelleted at 3,220*g* for 10 min, followed by four washing steps with PBS buffer. The final aggregates were resuspended and vortexed for 30 min, and then sonicated for 10 s followed by 10 s of rest time for a total of 30 s sonication time. The freshly sonicated samples were incubated on ice for 5 min and then used as seeds the seeding experiments. Several complementary seeding assays were performed; the first involving a previously described htt exon1-seeding protocol^50^ that measures the effects of the seeds on the aggregation kinetics of our fusion protein upon trypsin cleavage. The protease cleavage was performed for 10 min on ice at a protease/substrate molar ratio of 1:3, after which the reaction was quenched with phenylmethylsulfonyl fluoride inhibitor/substrate molar ratio of 150:1. The reaction mixture was split into three samples. To two of the samples 20 mol-% of pre-aggregated seeds were added. Next, the volume of all samples was adjusted with PBS to obtain a polyQ monomer concentration of 11.6 μM, after which aggregation was done at 22 °C. For each sample the aggregation progression was monitored using a combination of ThT assays (in duplicate or triplicate; see below) and a complementary HPLC-based assay, which detect the aggregated and monomer protein, respectively. These methods are described below. Complementary seeding assays probed the effect of the 20 mol-% of the exon1 fibril seeds on the aggregation at 37 °C of htt^NT^Q_23_P_10_K_2_ peptide. The htt^NT^Q_23_P_10_K_2_ was prepared and disaggregated^21^ in 1:1 (v/v) mixture of trifluoroacetic acid (TFA) and hexafluoroisopropanol overnight. The buffer was evaporated off under a N_2_ stream and the peptide was dried under vacuum for 1 h. The residue was dissolved in H_2_O adjusted to pH 3 with TFA. Residual aggregates were removed by ultracentrifugation at 386,000*g* for 1 h, after which the pH was adjusted to 7.0 using 10 × PBS buffer that was subsequently diluted 10-fold. Next, the seeds were added and the reaction kinetics monitored (in presence and absence of seeds) using ThT and HPLC-based assays, described below.

### ThT fluorescence assays

Aggregates were resuspended by aspiration and aliquots were diluted into a ThT stock solution (5 μM ThT, 10 mM sodium phosphate, 150 mM NaCl, pH 7.0). Samples were excited at 445 nm and the emission was recorded at 489 nm over several seconds on a FluoroMax-4 spectrofluorometer (Horiba; Kyoto, Japan). The excitation and emission slits were 2 and 4 nm, respectively. The ThT assays on the seeded aggregation at 22 °C starting with the htt exon1 fusion protein were performed in duplicate, except for the following measurements performed in triplicate: 22 °C seeds: 15, 45, 75, 105, 195 and 735 min.; 37 °C seeds: 15, 45, 75 and 135 min. The corresponding ThT measurements of the unseeded aggregation were performed in duplicate, except for trial 1's first four points (which were measured in triplicate) and its final time point that was measured once. The ThT assays of the seeded aggregation at 37 °C of the htt^NT^Q_23_P_10_K_2_ peptide were based on duplicate measurements, except for triplicate measurements of the 1,200, 1,740 and 2,700 min time points (37 °C seeds), and single measurements at 60 min (both seeds). The corresponding ThT assays of the unseeded material were performed in duplicate, except for 2,700 min time point that was measured in triplicate and the 60 min point that was measured once.

### HPLC-based sedimentation assay

As a complementary assay to the ThT assays of seeded and unseeded aggregation, we also performed a single measurement each of the peptide monomer concentration using an established HPLC-based sedimentation assay^21^,^63^. To do so, an aliquot was removed from the reaction mixtures at the indicated time points, and the solid material was pelleted at 20,800*g* for 15 min. The supernatant was diluted 2 × in formic acid, and loaded on an Agilent Zorbax SB-C8 4.6 × 50 mm column (1.8 μm particle size) using an analytical HPLC (Agilent Technologies). The monomer was eluted over a 15–35% gradient of acetonitrile in water, with 0.05% TFA at 37 °C. The elution of the monomer species was monitored by absorbance at 215 nm (A_215_), as it has no significant absorbance at A_280_. The relative amount of monomer in each sample was determined by integration of A_215_ peaks using the manual integration analysis mode with a manually defined linear baseline correction as provided by the ChemStation for the LC systems software (Agilent Technologies). To estimate the error in this manual integration and baseline correction, three independent integrations were applied to each datapoint, yielding an approximate measurement error of 5% in most of the measured values (see individual error bars in Fig. 8).

### Neuronal toxicity assays

Htt exon1 aggregates were prepared at 22 and 37 °C, as described above, at concentrations of 14.5 and 10.1 μM, respectively. The resulting fibrils were examined by negative stain TEM to verify the generation of the wide and narrow fibril polymorphs. After 1 week, the aggregated material was resuspended by aspiration followed by vortexing and shaking for 30 min. The remaining material was pelleted at 3,220*g* for 10 min, and the soluble MBP was removed by buffer exchange three times into PBS (0.02% w/v NaN_3_). After the samples were thoroughly resuspended, they were sonicated as described above. Before use in the toxicity assays, the fibrils were washed three times with PBS buffer and sonicated. The employed LUHMES cells are human-differentiated neurons derived from a clone of MESC2.10 cells. These human dopaminergic neurons were extensively studied and validated previously^51^,^52^,^53^,^64^. The employed cells were kindly provided to us by Professor Marcel Leist (University of Konstanz, Germany), and were used without further authentication. The cells were tested for mycoplasma contamination and were found to be mycoplasma-free. Non-differentiated LUHMES cells were grown in coated plates with poly-l-lysine (0.1 mg ml^−1^) in growth medium (DMEM/F12 medium supplemented with 1% N2-supplement (Life Technologies, Carlsbad, CA, USA), 100 U ml^−1^ penicillin, 100 mg ml^−1^ streptomycin and 0.04 μg ml^−1^ basic fibroblast growth factor (R&D Systems, Minneapolis, MN, USA)). For the differentiation experiments, LUHMES cells were grown in coated plates with poly-L-lysine (10 μg ml^−1^) followed by fibronectin (5 μg ml^−1^). Dopaminergic neurons were differentiated in DMEM/F12 medium with 1% N2-supplement, 1 μg ml^−1^ tetracycline, 100 U ml^−1^ penicillin, 100 mg ml^−1^ streptomycin, 0.49 mg ml^−1^ dibutyril cyclic AMP (Sigma-Aldrich) and 2 ng ml^−1^ glial cell-derived neurotrophic factor^52^,^53^. Following 6–7 days of *in vitro* differentiation, LUHMES cells expressed the dopamine transporter, the vesicular monoamine transporter 2, tyrosine hydroxylase and the neuronal form of *β*-III tubulin^53^,^65^. The immortalized mouse hippocampal HT-22 cells were cultured in Dulbecco's modified Eagle's medium with the addition of 10% heat-inactivated fetal calf serum, 100 U ml^−1^ penicillin, 100 mg ml^−1^ streptomycin and 2 mM glutamine. Toxicity measurements were performed by administering the sonicated fibrils at the indicated protein concentrations within the growth medium. After 24 and 48 h of treatment, quantification of cell viability was performed via a MTT (3-(4,5-dimethyl-2-thiazolyl)-2,5-diphenyl-2H-tetrazolium bromide) reduction assay at 0.5 mg ml^−1^ for 1 h. The reaction was terminated by removing the MTT solution and freezing the plate at −20 °C for at least 1 h. DMSO solvent was added to each well for 30 min under shaking conditions at 37 °C. The absorbance of each well was determined with a Synergy H1 Multi-Mode reader (Biotek, LA) at 570 nm with a reference filter at 630 nm (refs 52, 53).

### MAS ssNMR spectroscopy

Unless specified otherwise, MAS ssNMR experiments were performed using a wide-bore Bruker Avance I NMR spectrometer operating at ^1^H Larmor frequency of 600 MHz (14.1 Tesla) and triple-channel (HCN) Bruker 3.2 mm MAS NMR probes. Isotopically labelled samples were prepared and packed into 3.2 mm MAS rotors using an ultracentrifugal packing device^15^,^62^. CP-based peak assignments were obtained using 2D ^13^C–^13^C spectra employing DARR^66^ mixing, as well as standard heteronuclear 2D MAS ssNMR assignment spectra. Dynamics were probed via dipolar-recoupling curves based on a series of 2D PDSD experiments, with mixing times ranging from 0, 15, 50, 100, 250 to 500 ms. 2D peak volumes were integrated using the Gaussian peak fitting routines of the Sparky NMR software package, and normalized relative to corresponding diagonal peak volumes at zero mixing. The errors in the measured peak intensity were estimated based on the noise peak intensities in the spectra. ^1^H–^13^C dipolar couplings were probed via DIPSHIFT experiments^49^ using a 3.2-mm triple-channel HCN MAS probe in a wide-bore 750 MHz spectrometer from Bruker Biospin acquired via NIH S10 grant OD012213-01. The DIPSHIFT experiments employed a R18_1_^7^ pulse sequence^67^,^68^, at 10 kHz MAS and 277 K. We measured 12 increments constituting a 100 μs rotor period each, up to a maximum recoupling time of 1.1 ms. ^1^H–^13^C dipolar recoupling in the DIPSHIFT experiment was enabled by application of a R18_1_^7^ pulse sequence on the ^1^H channel at a 91 kHz RF power level. The initial ^13^C signal was generated with CP, using a 1.5 ms contact time. Highly mobile segments of the aggregated exon1 were identified using scalar-based spectroscopy employing refocused INEPT ^1^H–^13^C transfers combined with ^13^C–^13^C transfers using P9^1^_3_ TOBSY^69^,^70^,^71^. Water-exposure measurements were performed using ssNMR experiments in which rigid ^1^H signals were suppressed by T_2_ relaxation filtering, after which ^1^H–^1^H diffusion facilitated transfer of the remaining polarization of mobile solvent protons back into the immobilized protein assemblies^13^,^15^. The resulting polarization buildup in the protein residues was then monitored via 1D ^1^H–^13^C CP spectroscopy. 2D ^1^H–^13^C spectra were used to verify the origin of the (mobile) ^1^H polarization being the aqueous solvent. T_2_-filtered 2D ^13^C–^13^C DARR spectra were used to verify the identity of dominant peaks in the T_2_-filted 1D spectra.

Experimental details for all spectra are listed in Supplementary Table 2. ^1^H decoupling during acquisition and evolution periods was done with two-pulse phase modulation^72^, and MAS spinning rates were typically between 8.3 and 13 kHz (see Supplementary Table 2). Spectra were acquired using the Bruker Topspin software, processed using NMRPipe^73^. Chemical shifts were assigned and analysed using the Sparky and CcpNmr Analysis software packages^74^. Peak intensities were measured in the Bruker's Topspin software and CcpNmr Analysis, with the error in the intensities evaluated based on the noise peaks present in empty spectral regions. Numerical simulations of the DIPSHIFT experiments were performed with the SpinEvolution programme^15^,^75^. Chemical shift referencing to 4,4-dimethyl-4-silapentane-1-sulfonic acid (for ^13^C) was performed by indirect referencing via the ^13^C signals of adamantane^15^. Secondary shift calculations were done using published random coil shifts^76^.

### Data availability

Chemical shifts of the synthetic HNTF peptide fibrils (htt^NT^Q_30_P_10_K_2_) were reported previously^15^ and are accessible in the Biological Magnetic Resonance Data Bank (BMRB) as entry 25146. Assigned shifts of the mutant htt exon1 fibrils are available in Supplementary Table 3, and in the BMRB as entry 27045. UniProt entry P42858 has been used in this study. All other data are available from the corresponding author upon reasonable request.

## Additional information

**How to cite this article:** Lin, H.-K. *et al.* Fibril polymorphism affects immobilized non-amyloid flanking domains of huntingtin exon1 rather than its polyglutamine core. *Nat. Commun.* **8**, 15462 doi: 10.1038/ncomms15462 (2017).

**Publisher's note:** Springer Nature remains neutral with regard to jurisdictional claims in published maps and institutional affiliations.

## Supplementary Material

Supplementary Information Supplementary Figures, Supplementary Tables and Supplementary References.
