# Supplementary material for: Fibril polymorphism affects immobilized non-amyloid flanking domains of huntingtin exon1 rather than its polyglutamine core
Source: Nat Commun. 2017 May 24;8:15462. doi: 10.1038/ncomms15462 (PMC5458082; doi:10.1038/ncomms15462)
Supplement: Supplementary Information — Supplementary Figures, Supplementary Tables and Supplementary References. [file ncomms15462-s1.pdf]

Supplementary Table 1 – Theoretical and experimental molecular masses for the fusion protein and its cleaved products. *Samples of purified uncleaved fusion protein and freshly cleaved (using factor Xa) cleavage products were analyzed by electrospray ionization time-of-flight (ESI TOF) mass spectrometry (MS). Tabulated values are for unlabeled (natural abundance) protein. ESI TOF MS analysis of the U-^{13}C,^{15}N-labeled protein batches aggregated at room temperature and 37 °C indicated 97.5 and 97.8% labeling levels, respectively.*

**Theoretical**	**Fusion Protein [a] (Da)**	**MBP tag (Da)**	**Exon1 (Da)**
MBP-exon1 fusion construct from Hoop et al (2014) Biochemistry			
Theoretical	56283.1	42481.9	13819.2
ESI TOF	56283.7	42486.8	13817.3
Codon-optimized fusion construct reported here			
Theoretical	56684.5	42883.3	13819.2
ESI TOF	56688.5	42885.4	13818.4

[a)] The mass of the fusion protein is 18 Da larger than the cleaved MBP tag + Htt Exon1 due to the addition of H_2O in the hydrolysis cleavage reaction.

Supplementary Table 2 - Experimental conditions of ssNMR experiments. *Abbreviations: NS, number of scans per t_1 point; Temp., temperature; MAS, magic angle spinning rate; RD, recycle delay; TPPM, ^{1}H decoupling power during evolution and acquisition using the two-pulse phase modulation scheme; Mixing, ^{13}C-^{13}C or ^{1}H-^{1}H mixing time (ms); t_1 evol., maximum t_1 evolution time expressed in number of t_1 points (real+imaginary) x t_1 increment time.*

Fig. a)	Sample b)	Experiment	NS	Temp (K)	MAS (kHz)	RD (s)	TPPM (kHz)	t_1 evol. (μs)	Mixing (ms)
3a	*Htt exon1 fibrils; 37 °C*	^{1}H-^{13}C CP	*1024*	275	10	*2.8*	*83*	N/A	N/A
3b; S4d	*Htt exon1 fibrils; 22 °C*	^{1}H-^{13}C CP	*1024*	275	*13*	*2.8*	*83*	N/A	N/A
3d; S4b	*Htt exon1 fibrils; 37 °C*	^{13}C DP	*1024*	275	10	*2.8*	*83*	N/A	N/A
3e; S4a,e	*Htt exon1 fibrils; 22 °C*	^{13}C DP	*1024*	275	10	*2.8*	*83*	N/A	N/A
3g; S4c	*Htt exon1 fibrils; 37 °C*	^{1}H-^{13}C INEPT	*1024*	275	10	*2.8*	*83*	N/A	N/A
3h	*Htt exon1 fibrils; 22 °C*	^{1}H-^{13}C INEPT	1024	275	10	*2.8*	*83*	N/A	N/A
4a; S7	*Htt exon1 fibrils; 22 °C*	2D ^{13}C-^{13}C DARR	*325*	275	13	*2.8*	*83*	*724x27.6*	*15*
4b	*3mg HNTF peptide fibrils; A10, F11, L14, Q18-labeled*	2D ^{13}C-^{13}C DARR	*32*	*273*	*10*	*3.5*	*83*	*550x22.07*	*25*
4b	*2mg HNTF peptide fibrils; A2, L7, F17-labeled*	2D ^{13}C-^{13}C DARR	*64*	267	*16*	*3.5*	*83*	*480x20.83*	25
5a	*N-acetyl-Val-Leu crystals; 9% ^{13}C,^{15}N-labeled*	2D ^{13}C-^{13}C PDSD	*8*	275	*13*	*2.8*	*83*	*526x33.11*	0, 15, 50, 100, 250, 500
5b-d; S5a-f	*Htt exon1 fibrils; 22 °C*	2D ^{13}C-^{13}C PDSD	*96*	275	*13*	*2.8*	*83*	*526x33.11*	0, 15, 50, 100, 250, 500
6a; S7	*Htt exon1 fibrils; 22 °C*	2D ^{13}C-^{13}C INEPT-TOBSY	*96*	275	*8.333*	*2.6*	*83*	*338x83.33*	*6*
6b	*Htt exon1 fibrils; 37 °C*	2D ^{13}C-^{13}C INEPT-TOBSY	*96*	275	*8.333*	*2.6*	*66*	*338x83.33*	*6*
7a	*Htt exon1 fibrils; 37 °C*	^{1}H-^{13}C CP	*1024*	275	*13*	*2.8*	*83*	N/A	N/A
7a	*Htt exon1 fibrils; 37 °C*	T_2 filtered	*1024*	275	*13*	*2.8*	*83*	N/A	7
7b	*Htt exon1 fibrils; 22 °C*	^{1}H-^{13}C CP	*1024*	275	*13*	*2.8*	*83*	N/A	N/A
7b	*Htt exon1 fibrils; 22 °C*	T_2 filtered	*1024*	275	*13*	*2.8*	*83*	N/A	7
S4f	*Htt exon1 fibrils; 22 °C*	^{1}H-^{13}C CP	*1024*	275	*13*	*2.8*	*83*	N/A	N/A
S4g	*Htt exon1 fibrils; 22 °C*	^{13}C DP	*1024*	275	*13*	*2.8*	*83*	N/A	N/A
S6	*Htt exon1 fibrils; 22 °C*	DIPSHIFT c)	*256*	277	*10*	*4.0*	*83*	*12 x 100*	*0-1.1ms*
S6	*Htt exon1 fibrils; 37 °C*	DIPSHIFT c)	*256*	277	*10*	*4.0*	*83*	*12 x 100*	*0-1.1ms*

a) Supplementary Figure X is listed as Fig. SX. Various Supplementary data are extended representations of data summarized in the main figures.
b) The fibrils are U-^{13}C,^{15}N-labeled, unless indicated otherwise; the 22°C and 37 °C htt exon1 fibrils contain 4.4 and 1.1 mg protein, respectively.
c) This experiment was performed on a 750 MHz (^{1}H) spectrometer.

Supplementary Table 3 - ^{13}C chemical shifts of assigned residues in htt exon1 fibrils*. The standard deviation of the ^{13}C chemical shifts is ± 0.1-0.3 ppm based on the systematic comparison, during the peak assignment process, of peak positions between different spectra reported here (Supplementary Table 2) and in prior work [1,2]. No significant chemical shift differences were apparent between the different fibril types. The final columns indicate whether these signals were observed to be in immobilized or rigid parts of the fibril ("Visible by CP"), in the dynamic, solvent-exposed regions ("Visible INEPT"), or in both.*

	C'	Cα	Cβ	Cγ	Cδ(1)	Cδ2	Cε/Cζ	Visible by CP	Visible INEPT
M1			34.5	25.3			17.0		++
A2	177.5	52.6	19.4					+	+
T3		70	69	21.5				+	+
L4		57.5	40.9	26.3	23.7			+	
E5		58.9	29.0	36.4	183.5			+	
K6	179.6	59.5	32.6	25.8	29.3		42.3	+	
L7		57.9	41.7	26.9	23.7			+	
A10		55.0	18.2					+	
Gln 'a'	175.9	56.1	34.2	34.2	178.6			++	-
Gln 'b'	174.1	53.9	31.7	30.2	177.6			++	-
Gln 'c'	--	--	--	33.9	180.3			+	+
Pro RC	177.0	63.0	32.1	27.5	50.3			+	++
Pro PPII	-	61.2	30.7	27.4	50.2			++	+
V103		61.8	33					+	+
E105	175.1	56.2	30.6	36.4	184.1				+
E106		56	29.8	36.1	184.1				+
R110			30.3	27	43.0				+

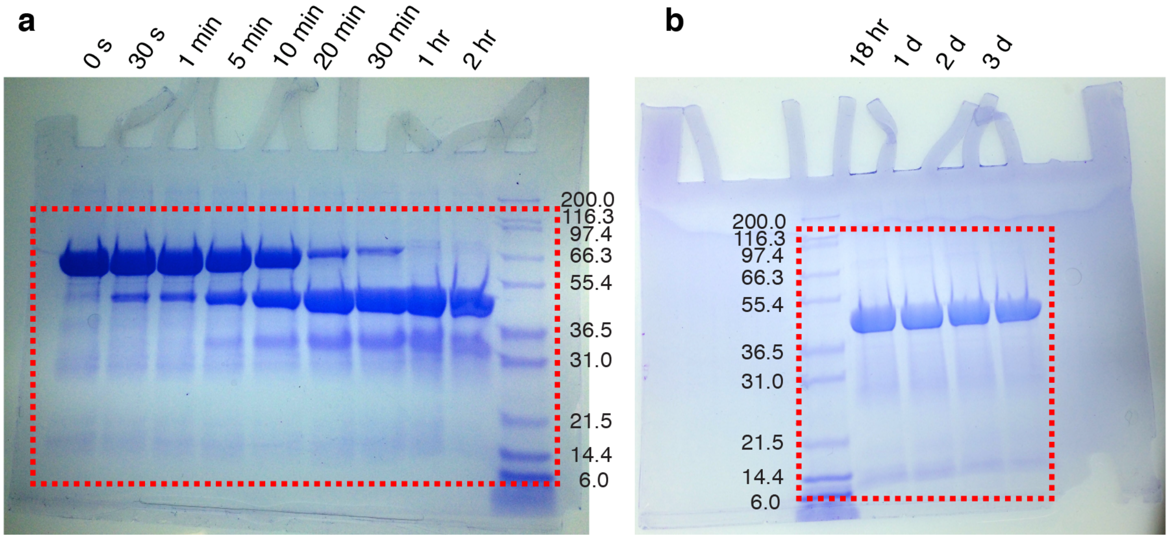

Supplementary Figure 1. *Full versions of SDS PAGE gels. (a) SDS PAGE gel from Figure 2a. Dashed red box marks the area shown in the main figure. (b) SDS PAGE gel from Figure 2a, with dashed red box showing area from main text figure.*

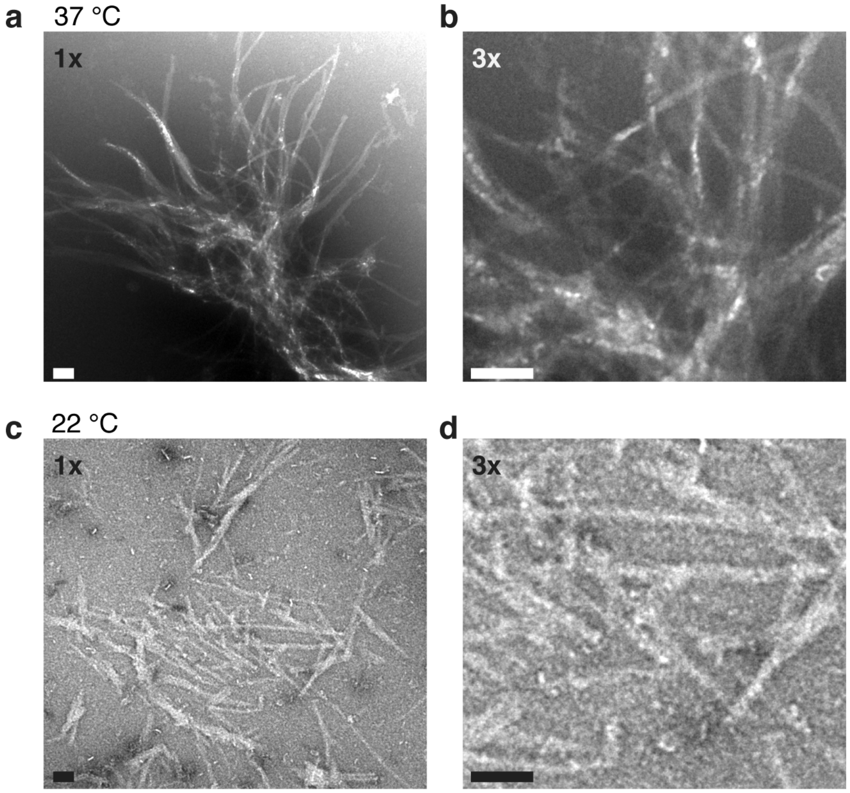

Supplementary Figure 2. *TEM data comparing polyQ-expanded htt exon1 fibrils formed at 22 and 37 °C. (a,b) Negative stain TEM of htt exon1 fibrils formed at 37°C, at two levels of magnification. (c-d) Negative stain TEM of htt exon1 fibrils formed at 22°C, at two levels of magnification. All scale bars are 100 nm.*

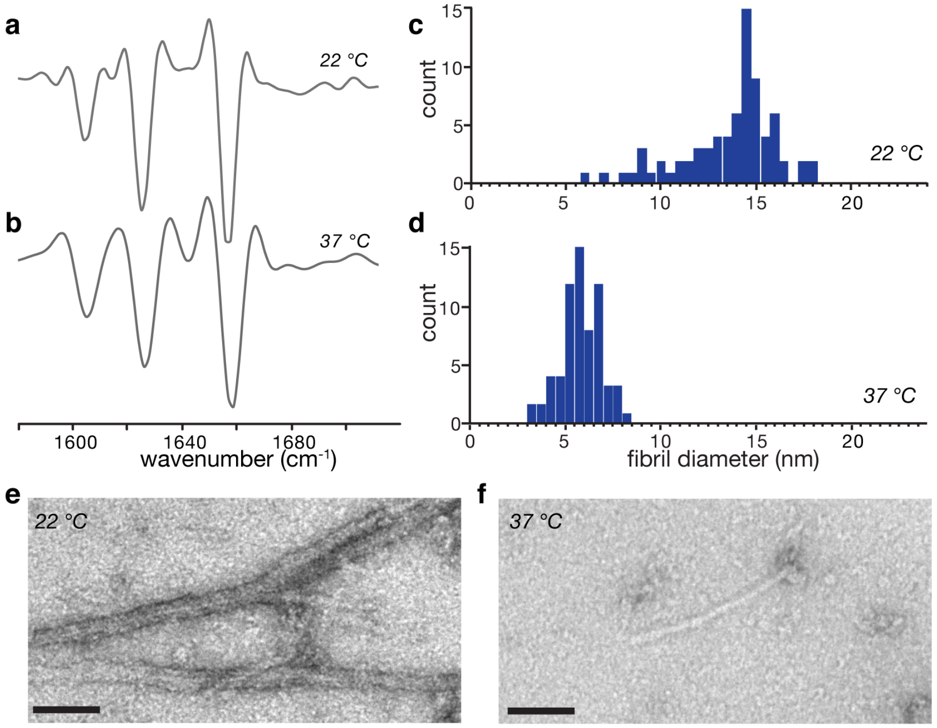

Supplementary Figure 3. *FTIR and TEM results for additional independently prepared polymorphic htt exon1 aggregates formed at 22 °C and 37 °C. (a) Second-derivative FTIR spectrum for htt exon1 fibrils prepared at room temperature (22 °C) and (b) 37 °C. (c,d) Histograms of the TEM-based fibril width measurements for these same preparations. The fibrils formed at 22 °C and 37 °C have a fiber diameter of 15.2 ± 3.5 nm and 6.4 ± 0.9 nm, respectively (s.d.; reflecting 111 and 66 measurements, each). (e,f) Representative negative stain TEM micrographs for the two fibril samples. Scale bars are 100 nm.*

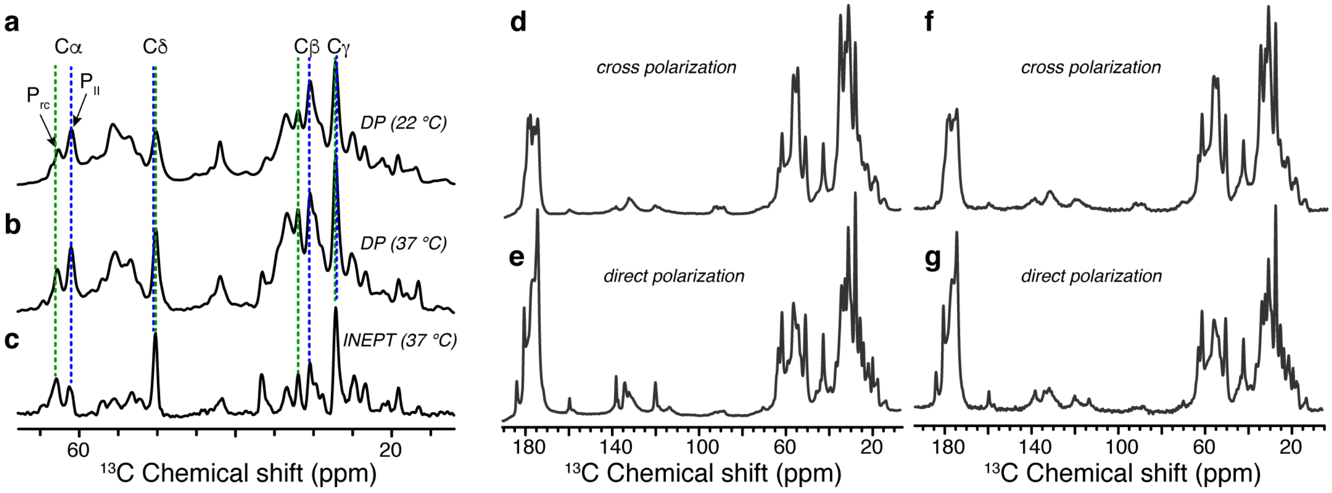

Supplementary Figure 4. *Additional 1D MAS NMR spectra comparing different fibril preparations. (a) Comparison of the 1D ^{13}C direct polarization (DP) spectrum of fibrils formed at 22 °C and (b) 37 °C, showing the relative overall populations of the two Pro signals. (c) The ^{1}H-^{13}C INEPT 1D spectrum that shows only the highly dynamic residues in the 37 °C fibrils. The IDP-like Pro make up the majority of the dynamic PRD residues, but there is also a substantial signal from dynamic PPII helices. (d-g) Reproducibility of the CP and DP spectra of two independently prepared fibrils formed at 22 °C: (d-e) the same sample as in Fig. 3; (f-g) fibrils prepared independently at 22 °C out of a mixture of ^{13}C-only and ^{15}N-only labeled htt exon1 (see also ref. [2]).*

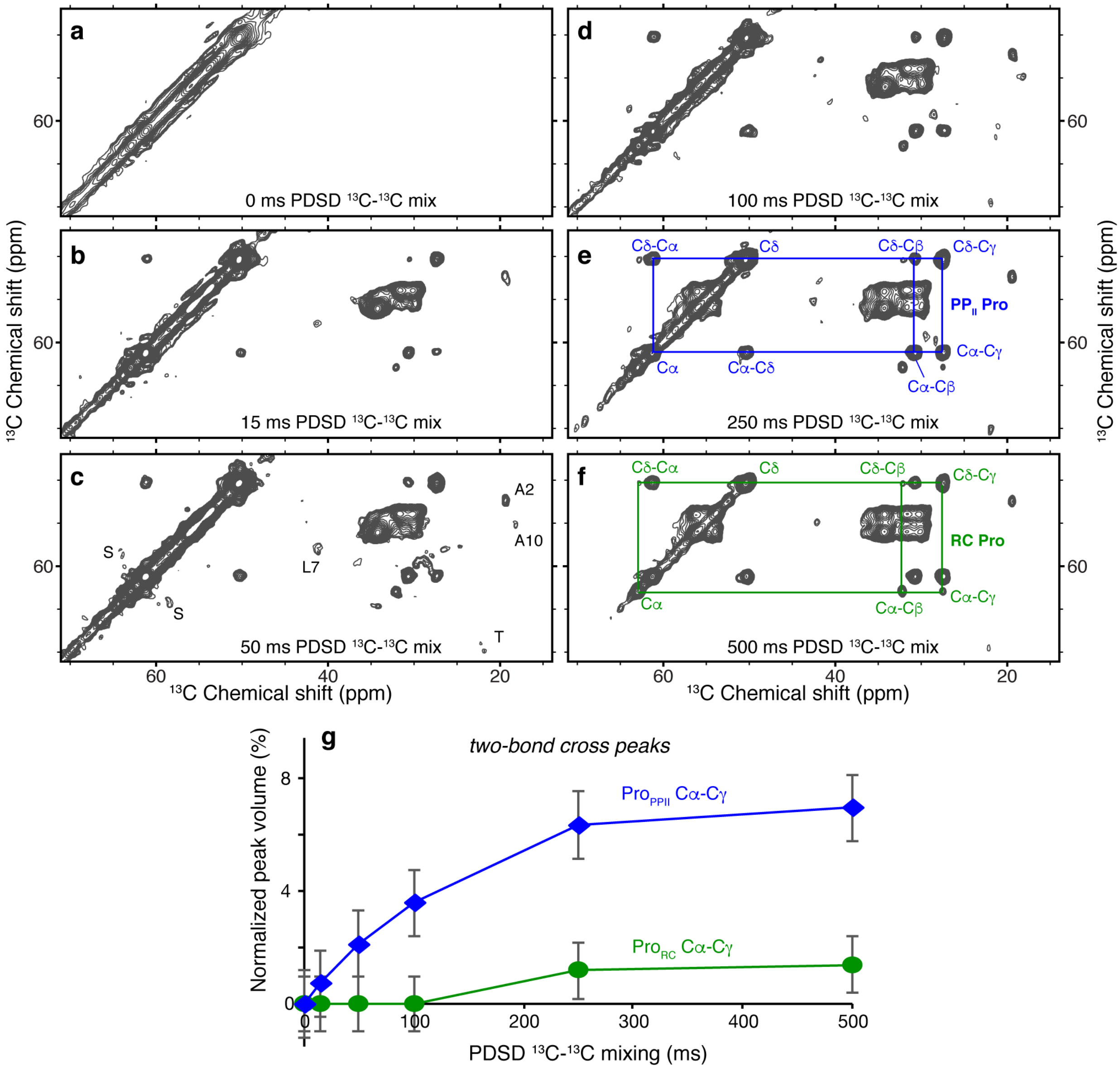

Supplementary Figure 5. *Relative dynamics and populations of the PPII and IDP-like Pro. (a-f) 2D regions from ^{13}C-^{13}C spectra with increasing PDSD mixing. Panels (c,e,f) include the assignments of PPII-structured Pro, random coil (RC) Pro, and selected other peaks. (g) PDSD buildup profiles for the two-bond Cα-Cγ distance within the Pro ring, comparing PPII Pro (blue; top) and RC Pro (green; bottom). The latter's lower buildup indicates reduced dipolar couplings and increased motion. Error bars indicate the s.d. in the integrated peak volumes, determined as described in the Methods.*

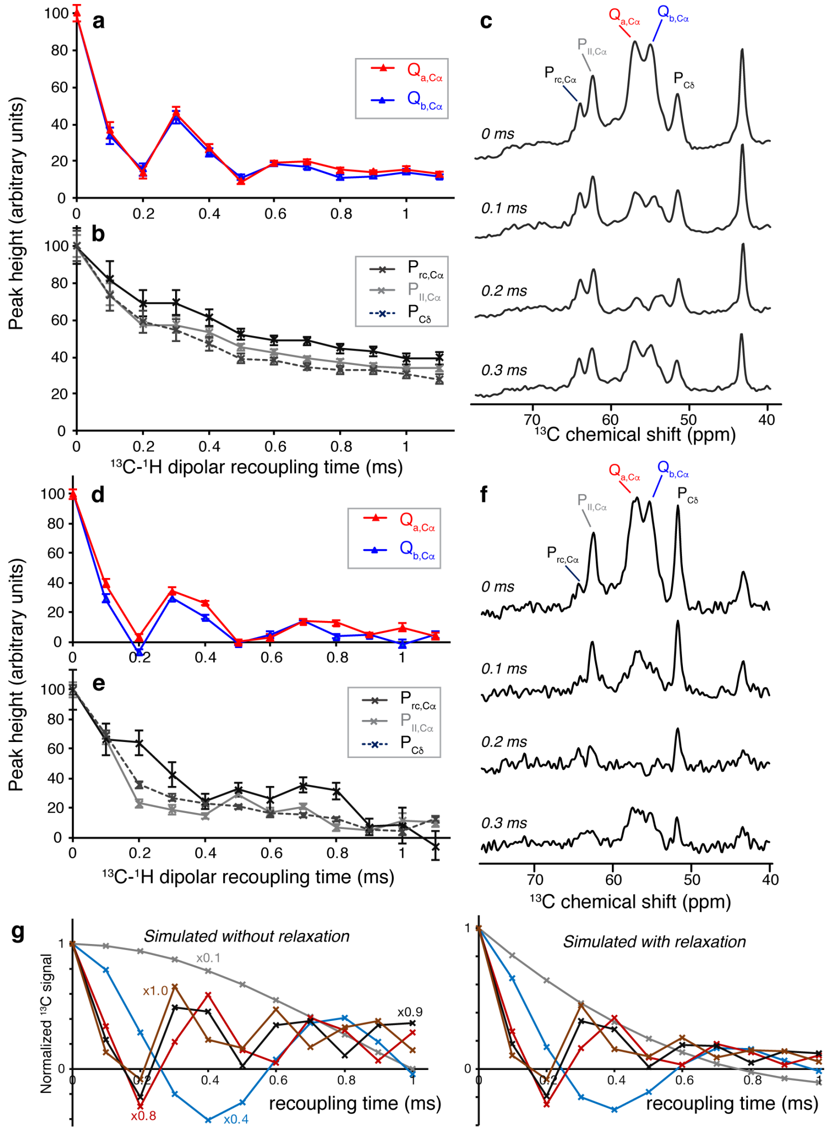

Supplementary Figure 6. *Dynamics-sensitive ^{13}C-^{1}H dipolar coupling measurements. Dipolar dephasing curves for Gln and Pro C-H bonds from a DIPSHIFT ssNMR experiment [3] with R18$_7^1$ ^{1}H-^{13}C dipolar recoupling [4,5]. (a) Dephasing curves for the Gln Cα of the two β-strand types (named 'a' and 'b') that form the polyQ amyloid core in the 22 °C fibrils. Rapid decay in first 200 μs and strong oscillations indicate strong ^{1}H-^{13}C dipolar couplings, and thus an absence of motion. (b) Analogous dephasing curves for the Cα of random coil (P_{rc}) and PPII-structured Pro (P_{II}). The curve for the combined Pro Cδ is also shown. All Pro dipolar dephasing occurs more slowly and lacks the oscillations of the rigid polyQ amyloid core, indicating the dynamics of the PRD flanking domain. (c) The ^{13}C 1D spectra of the first four points of the DIPSHIFT experiment, showing the clear differences in Gln and Pro Cα peak intensities as a function of the mixing time. (d-f) Corresponding data for the fibrils formed at 37 °C. These fibrils show a similar DIPSHIFT curves as the 22 °C fibrils, indicating rigid Gln backbones and dynamic Pro residues. The PPII helical Pro residues show a faster decay, due to being more restricted in their motion. All measurements were performed at 10 kHz MAS on a 750 MHz (^{1}H) ssNMR spectrometer. Error bars reflect s.d. in integrated peak volumes, as delineated in the Methods section. (g) Numerical simulations of the R18$_7^1$recoupling for different dipolar scaling factors (as marked), without relaxation (left) or with 0.5ms relaxation (right), performed as previously described [1]. A 1.0 scaling factor indicates a complete lack of motion, with decreasing values corresponding to increasing dynamics.*

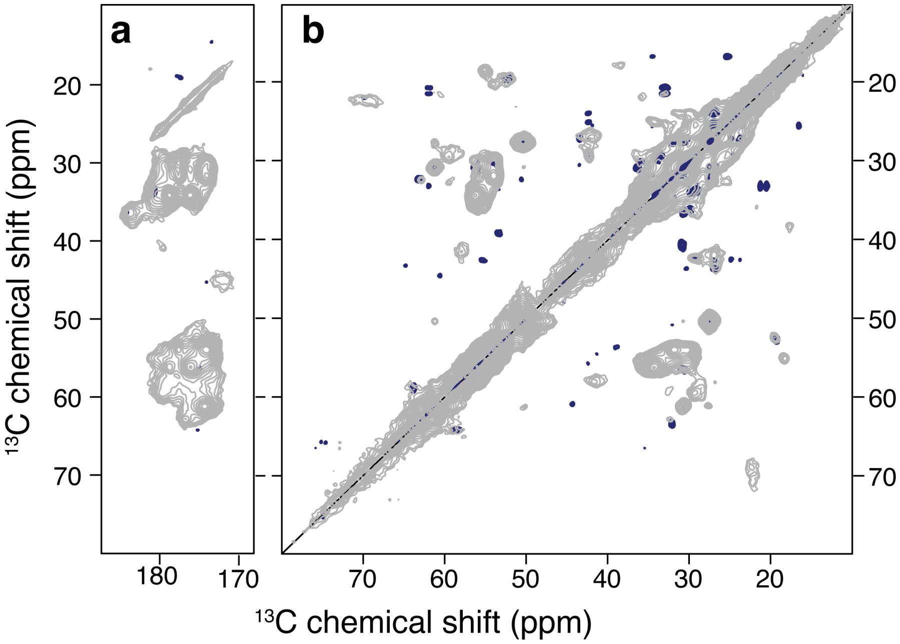

Supplementary Figure 7. *Overlay of INEPT- and CP-based 2D spectra of uniformly ^{13}C- and ^{15}N-labeled htt exon1 fibrils formed at 22 °C. (a) Carbonyl – aliphatic cross-peaks. (b) Aliphatic spectral region. The grey spectrum is the CP/DARR 2D spectrum from Fig. 4(a), which features only signals of the immobilized and rigid parts of the fibrils. The dark blue spectrum is the INEPT/TOBSY 2D from Fig. 6(a), and shows only signals from the most dynamic parts of the fibrils.*

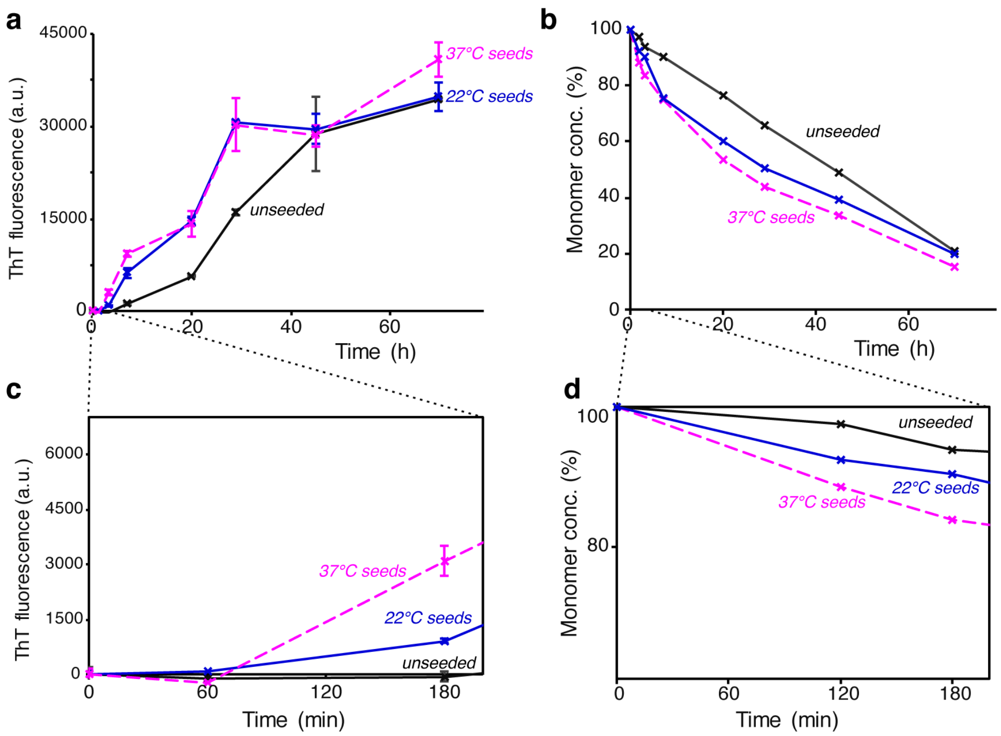

Supplementary Figure 8. *Additional seeding assay results. (a) Seeding effects on the aggregation of htt^{NT}Q$_{23}$P$_{10}$K$_2$ peptide[6] at 37 °C, monitored by ThT fluorescence measurements. Reactions were performed without seeds (black; solid), and with 20-mol-% htt exon1 seeds prepared either at 22 °C (dark blue; solid), or 37 °C (magenta; dashed). Error bars indicate s.d. with n=2 or n=3, as delineated in the Methods section. (b) In the same samples, sedimentation followed by reverse-phase HPLC was used to monitor the percent monomer remaining in the supernatant. (c,d) Enlargements of the first 200 minutes of panels (a) and (b), respectively. Both types of polymorphs are capable of cross-seeding the aggregation of this peptide featuring a shorter polyQ length that is below the HD threshold. In the earliest stages (see panels c,d) the fibrils formed at 37 °C appear to be more effective at seeding this aggregation reaction.*

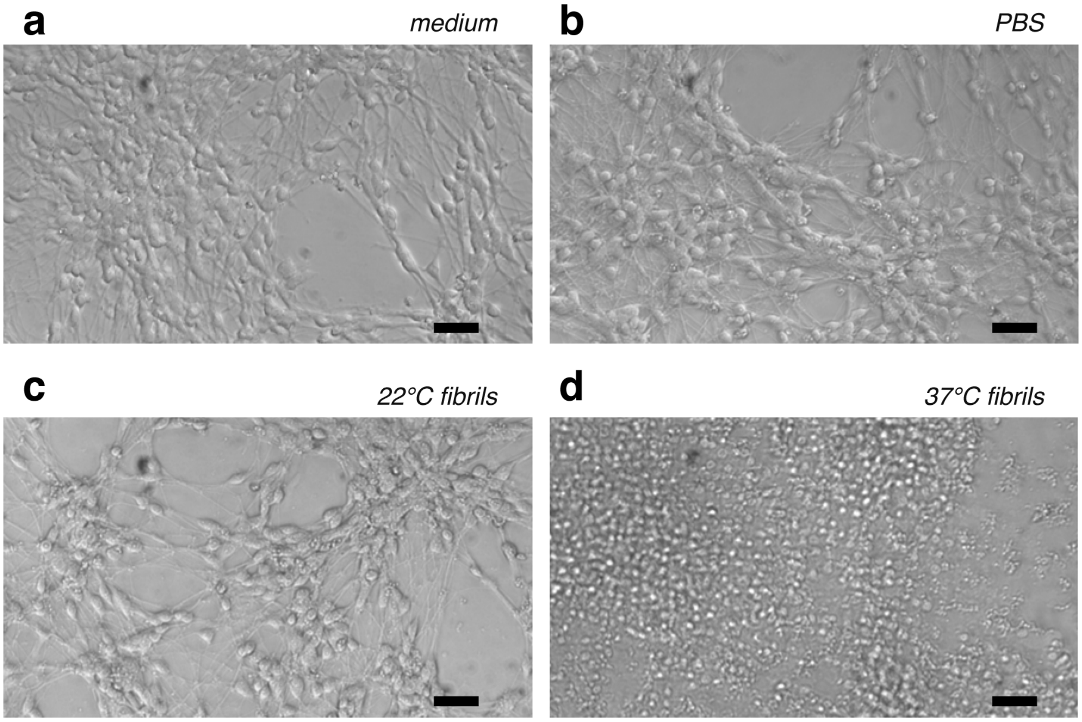

Supplementary Figure 9. *Microscope images of the treated and untreated human dopaminergic neurons. (a) Control neurons compared to treated neurons 24 h after administering (b) PBS buffer only, (c) 5 μM of the thick 22 °C fibrils in PBS, and (d) 5 μM of the thinner 37 °C fibrils in PBS. Scale bars are 50 μm.*

Supplementary References

1. Hoop, C.L. et al. Polyglutamine amyloid core boundaries and flanking domain dynamics in huntingtin fragment fibrils determined by solid-state nuclear magnetic resonance. *Biochemistry* **53**, 6653-6666 (2014).
2. Hoop, C.L. et al. Huntingtin exon 1 fibrils feature an interdigitated β-hairpin-based polyglutamine core. *Proc. Natl. Acad. Sci. U.S.A.* **113**, 1546-1551 (2016).
3. Munowitz, M., Griffin, R.G., Bodenhausen, G. & Huang, T.H. Two-dimensional rotational spin-echo nuclear magnetic resonance in solids: correlation of chemical shift and dipolar interactions. *J. Am. Chem. Soc.* **103**, 2529-2533 (1981).
4. Carravetta, M., Edén, M., Zhao, X., Brinkmann, A. & Levitt, M.H. Symmetry principles for the design of radiofrequency pulse sequences in the nuclear magnetic resonance of rotating solids. *Chem. Phys. Lett.* **321**, 205-215 (2000).
5. Zhao, X., Edén, M. & Levitt, M.H. Recoupling of heteronuclear dipolar interactions in solid-state NMR using symmetry-based pulse sequences. *Chem. Phys. Lett.* **342**, 353-361 (2001).
6. Thakur, A.K. et al. Polyglutamine disruption of the huntingtin exon 1 N terminus triggers a complex aggregation mechanism. *Nat. Struct. Mol. Biol.* **16**, 380-389 (2009).
